# Chemo‐Enzymatic Synthesis of Pyrazines and Pyrroles

**DOI:** 10.1002/anie.201810555

**Published:** 2018-11-21

**Authors:** Jin Xu, Anthony P. Green, Nicholas J. Turner

**Affiliations:** ^1^ School of Chemistry University of Manchester Manchester Institute of Biotechnology 131 Princess Street Manchester M1 7DN UK

**Keywords:** biocatalysis, nitrogen heterocycles, pyrazine, pyrrole, ω-transaminase

## Abstract

Herein we report the biocatalytic synthesis of substituted pyrazines and pyrroles using a transaminase (ATA) to mediate the key amination step of the ketone precursors. Treatment of α‐diketones with ATA‐113 in the presence of a suitable amine donor yielded the corresponding α‐amino ketones which underwent oxidative dimerization to the pyrazines. Selective amination of α‐diketones in the presence of β‐keto esters afforded substituted pyrroles in a biocatalytic equivalent of the classical Knorr pyrrole synthesis. Finally we have shown that pyrroles can be prepared by internal amine transfer catalyzed by a transaminase in which no external amine donor is required.

Amine transaminases (ATAs) have emerged in recent years as highly valuable biocatalysts for the conversion of a broad range of ketones and aldehydes to the corresponding primary amines.^1^ ATAs have found application especially in the synthesis of chiral amine building blocks for natural products, pharmaceuticals and agrochemicals and have been used for large scale manufacture of APIs (e.g. sitagliptin). ATA catalyzed reactions are reversible and hence strategies have been developed to ensure high conversion of carbonyl substrate to amine product including (i) use of excess amine donor, (ii) removal of products in situ, and (iii) use of “smart” amine donors to displace the equilibrium of the reaction.^2^ ATAs have also been extensively engineered to broaden substrate scope, enhance selectivity and improve stability under process conditions.^3^ However to date their application beyond the production of chiral amines has been limited. Herein we show that ATAs can be employed for the synthesis of N‐heteroaromatic compounds, specifically pyrazines and pyrroles, and demonstrate advantages of using biocatalysis over conventional methods for the preparation of these compounds.

N‐Heterocyclic motifs, both saturated and unsaturated, feature widely in pharmaceutical compounds and constitute privileged scaffolds.^4^ Amongst the plethora of different systems, pyrroles feature widely (e.g. Atorvastatin and Sunitinib, Figure 1 a)^5^ as well as substituted piperidines,^6^ pyrrolidines^7^ and indoles. Pyrazines are important components of aroma and flavor compounds^8^ including Polycartine B. Based upon a retrosynthetic analysis,^9^ we envisaged using ATAs to catalyze amination of α‐diketones into the corresponding α‐amino ketones which could then serve as key building blocks for both pyrazine (path a) and pyrrole (path b) synthesis (Figure 1 b). A key challenge in this approach is to be able to control pyrazine versus pyrrole synthesis by appropriate selection of reaction conditions.

**Figure 1:**
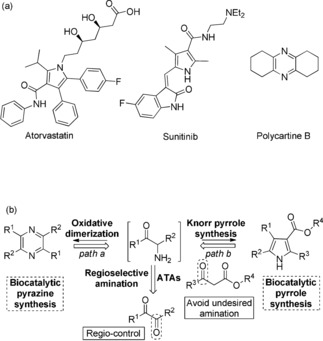
a) Two important drugs (Atorvastatin^5^ and Sunitinib^4^) containing pyrrole functional group and a pyrazine (Polycartine B^8^) used as flavor enhancer. b) A biocatalytic retrosynthetic approach to the synthesis of pyrazine and pyrrole mediated by ATAs.^9^

Initially two symmetrical α‐diketones, **1** and **2**, and one non‐symmetrical α‐diketone **3** were examined as substrates for ATA‐113 using either (*S*)‐aminotetralin (1.1 equiv) or isopropylamine (10 equiv) as amine donor (Scheme 1). All reactions proceeded smoothly with α‐diketones fully consumed (GC‐MS) after 72 h to yield pyrazine products (see the Supporting Information for GC‐MS and ^1^H NMR analyses of the reactions reported in this manuscript).

**Scheme 1:**
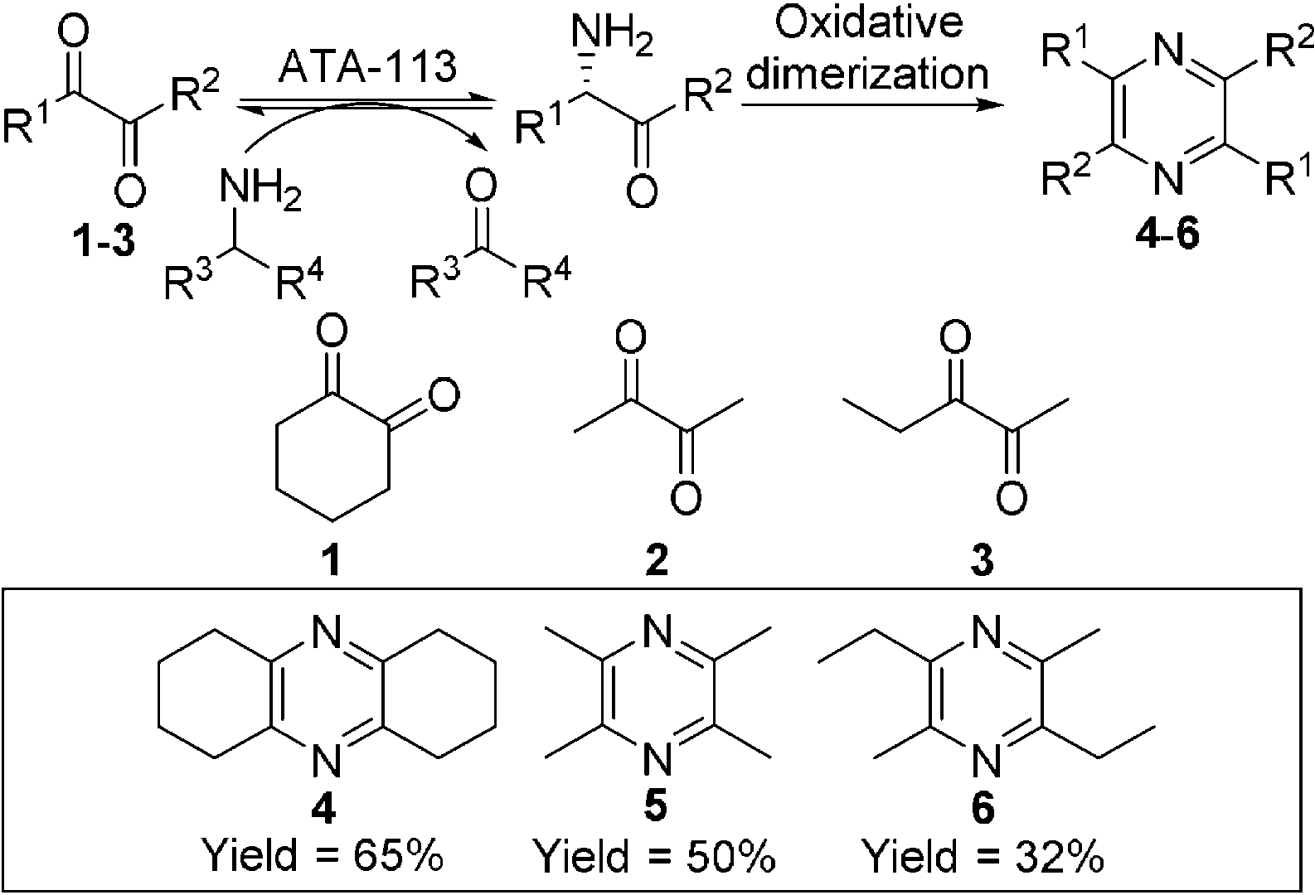
A chemo‐enzymatic approach for the synthesis of symmetric substituted pyrazines by employing (*S*)‐selective ATA‐113.

With isopropylamine as amine donor, pyrazine products were directly extracted from aqueous phase in pure form, with no significant by‐product or impurity observed. Pyrazines **4** and **5** are used as flavor enhancers8b and for the treatment of coronary atherosclerotic disease and ischemic cerebrovascular disease^10^ respectively. Pyrazine **6** was isolated as a single regioisomer, reflecting the selectivity of the biocatalytic amination step. Having established the viability of using ATAs for pyrazine synthesis, our attention turned to the Knorr pyrrole synthesis in which an α‐amino ketone is condensed with a β‐keto ester. A biocatalytic approach to pyrrole synthesis presents two key challenges: (i) the prevention of undesired oxidative dimerization of α‐amino carbonyl intermediates (pyrazine synthesis) and (ii) the chemo‐selective amination of α‐diketones in the presence of β‐keto ester co‐substrates.

During our initial studies, the products of amination of aryl‐substituted α‐diketones were observed not to spontaneously dimerize to pyrazines by GC‐MS analysis. Hence, aryl α‐diketone **7** was chosen as a candidate substrate to couple with β‐keto ester **a** (10 equiv). ATA‐117 catalyzed regioselective amination of α‐diketone **7** at C‐3 with high efficiency, the product of which underwent spontaneous coupling with β‐keto ester **a** to yield pyrrole **7 a** (Entry 1, Table 1) with no observable pyrazine formation. Reaction progress was determined by both GC‐MS analysis and ^1^H NMR analysis with the addition of 1,3‐dinitrobenzene (1 equiv) as internal standard (Table 1 a) for quantitative analysis of reaction components. The conversion of α‐diketone **7** in the presence of β‐keto ester **a** proceeded smoothly and after 72 h a 55 % conversion was observed via ^1^H NMR analysis. A preparative scale reaction yielded pyrrole **7 a** which was isolated in 38 % yield (Entry 1, Table 1). The generality of the process was investigated by examining a series of aryl substituted α‐diketones **7**–**12** in combination with two β‐keto esters **a** and **b** using ATA‐117 with (*R*)‐aminotetralin as amine donor (Table 1 b). All preparative scale reactions were determined to be successful by both GC analysis and ^1^H NMR analysis, with isolation of pyrrole products subsequently (Entries 2–7, Table 1).

**Table 1:** Conversion of aryl α‐diketones **7**–**12** to pyrroles **7**–**12 a** and **7 b** in the presence of β‐keto esters **a** and **b**. 
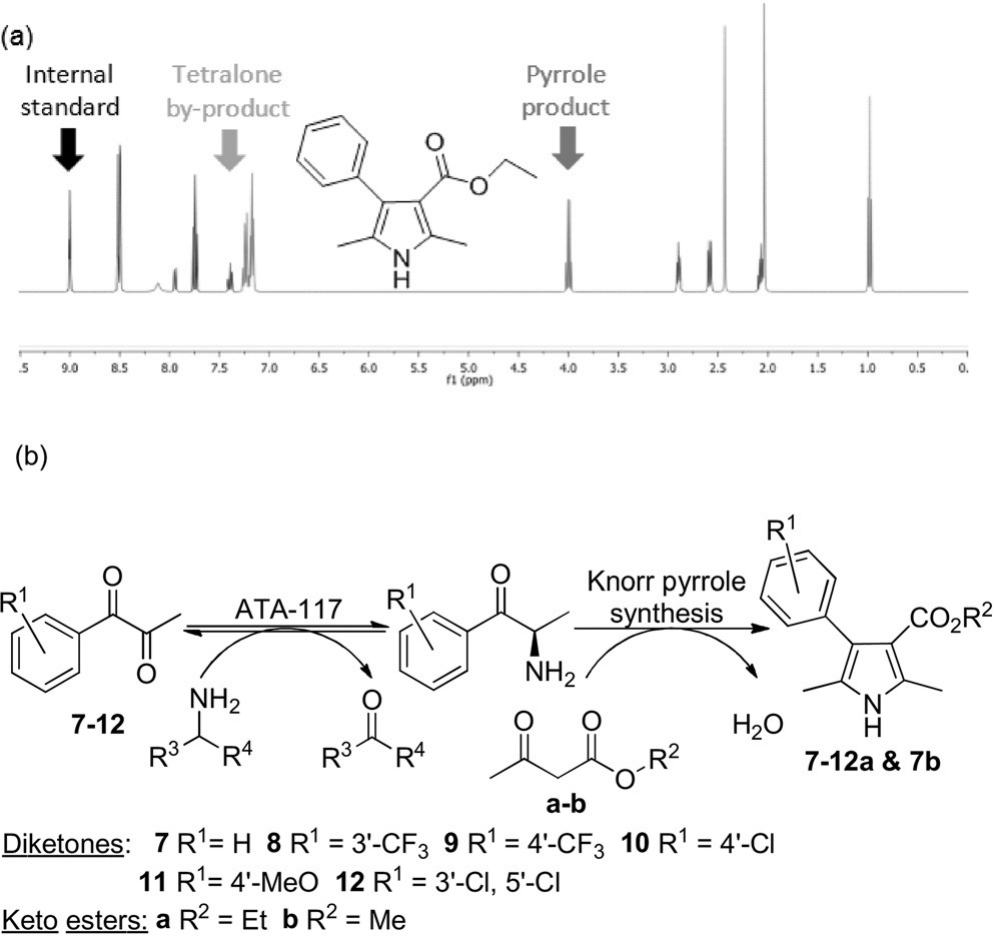

Entry	Product	R^1^	R^2^	NMR Conv. [%]^[a]^	Yield [%]
1	**7 a**	H	Et	55	38
2	**8 a**	3′‐CF_3_	Et	36	34
3	**9 a**	4′‐CF_3_	Et	26	21
4	**10 a**	4′‐Cl	Et	42	36
5	**11 a**	4′‐MeO	Et	60	31
6	**12 a**	3′‐Cl, 5′‐Cl	Et	78	48
7	**7 b**	H	Me	n.d.^[b]^	42

[a] Determined by ^1^H NMR analysis with the addition of 1,3‐dinitrobenzene (1 equiv) as internal standard after 72 h. [b] n.d.=not determined, pyrrole **7 b** directly isolated by flash chromatography after extraction.

Having established an effective means of synthesizing pyrroles from aryl α‐diketones, we next attempted the more challenging synthesis of pyrroles from dialkyl α‐diketones in which the corresponding α‐amino ketones are more susceptible to oxidative dimerization to form pyrazines).

Initially the simplest dialkyl α‐diketone **2** was chosen as the starting material to couple with β‐keto ester **b** and the reaction monitored by GC‐MS analysis. Using the original reaction conditions, the formation of both pyrazine **5** and pyrrole **2 b** was detected with an 81:19 pyrrole/pyrazine ratio (Entry 3, Table 2) and a 30 % yield of pyrrole product was obtained. Decreasing the pH of the reaction led to a higher pyrrole/pyrazine ratio (Entries 1–4, Table 2) with the pyrrole/pyrazine ratio (99:1) optimal at pH 5 (Entry 1, Table 2). Further optimization with reduced equivalents of β‐keto ester **b** at pH 5 (Entries 5–7, Table 2) yielded a pyrrole/pyrazine ratio (97:3) with 3 equiv of β‐keto ester **b** (Entry 5, Table 2). Even with 1 equiv of β‐keto ester **b**, the pyrrole/pyrazine ratio was still maintained at a high level (87:13) (Entry 7, Table 2).

**Table 2:** Conversion of dialkyl α‐diketone **2** to pyrazine **5** and pyrrole **2 b** in the presence of β‐keto ester **b**.^[a]^ 
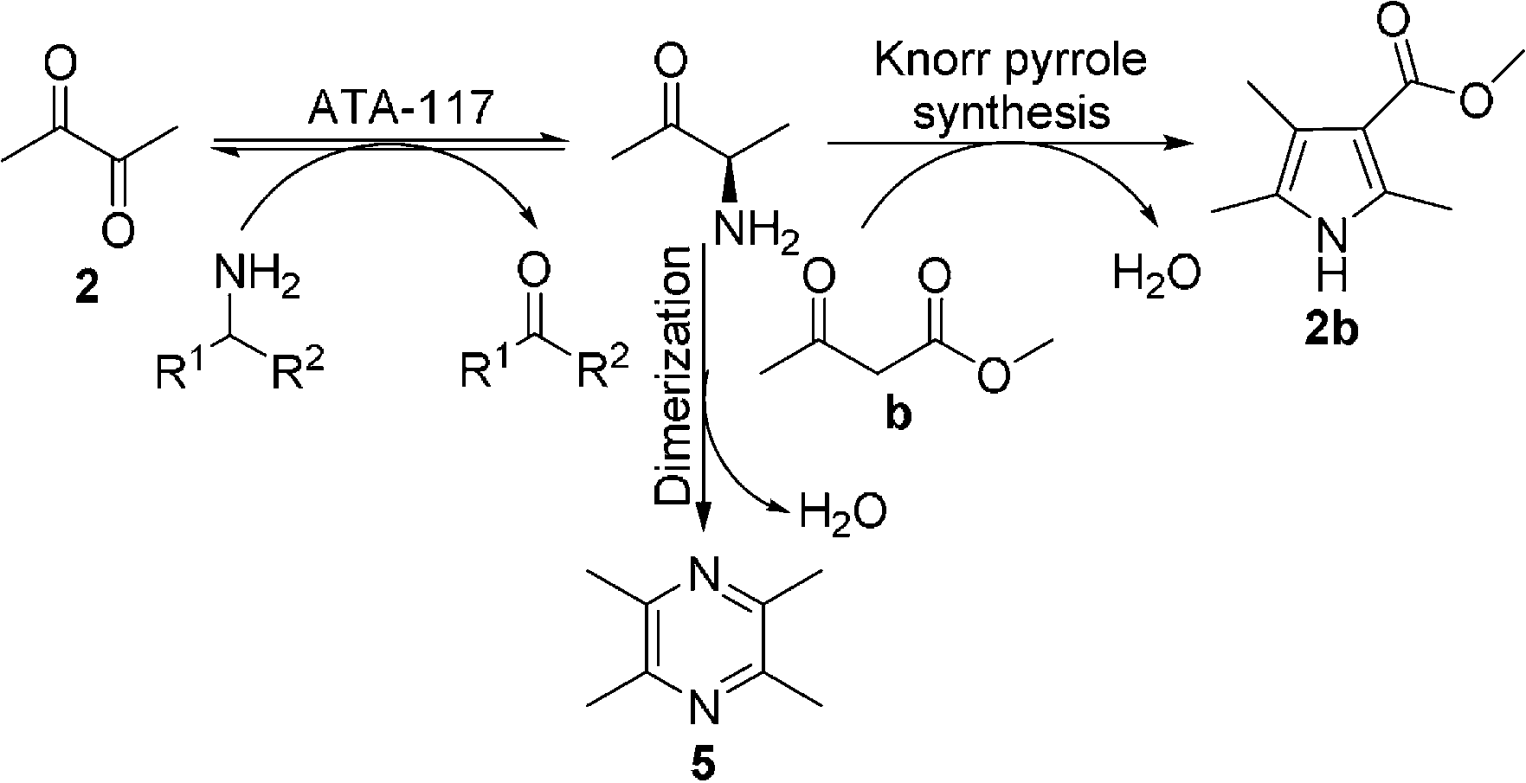

Entry	pH	Equiv of **b**	Pyrrole/pyrazine ratio [%]^[b]^
1	5	10	99:1
2	6	10	96:4
3	7.4	10	81:19
4	9	10	69:31
5	5	3	97:3
6	5	2	89:11
7	5	1	87:13

[a] Reaction condition: 20 mm α‐diketone **2** and 20–200 mm β‐keto ester **b**. [b] Determined by GC‐MS analysis after 24 h.

The ATA mediated synthesis of pyrroles relies on amination of the α‐diketone substrate in the presence of β‐keto ester, raising the question as to whether the ATAs is strictly selective for α‐diketones, or whether undesired amination of the β‐keto esters proceeds in a reversible fashion with the reaction driven by the thermodynamically favorable pyrrole formation. To probe the kinetic substrate selectivity, the reaction was initiated using the corresponding β‐amino ester **13** as substrate in the presence of α‐diketone but no additional amine donor. GC and ^1^H NMR analysis both confirmed pyrrole synthesis with a conversion of 58 % after 72 h. Under these conditions, the amino group of the β‐amino ester is transferred to α‐diketone **7** to give the corresponding α‐amino ketone precursor which then reacts with the β‐keto ester **a** to form pyrrole **7 a** via Knorr pyrrole synthesis (Scheme 2). Control reactions in the absence of ATA failed to produce any pyrrole product, confirming that the transformation is enzyme catalyzed. This internal amine transfer system provides an alternative disconnection for biocatalytic pyrrole synthesis exploiting the reversible nature of biocatalytic amination to shuttle amine functionality across reactions partners. Ideally, a stoichiometric ratio of β‐amino ester and α‐diketone would be consumed to access stoichiometric pyrrole product in the absence of external amine donor, while the only by‐product is water. Previously, a similar mechanism has been proposed to shuttle the amine functionality across the whole molecular framework between different carbonyl positions.^11^

**Scheme 2:**
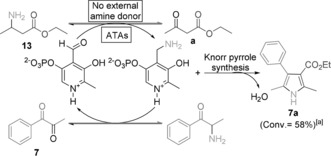
Proposed mechanism of amine functionality shuttle system for the biocatalytic synthesis of pyrrole **7 a** from aryl α‐diketone **7** and a self‐sufficient amine donor **13** employing ATAs. [a] Determined by ^1^H NMR analysis after 72 h.

In summary, we have developed biocatalytic methodology for the synthesis of pyrazines and pyrroles using transaminases. Pyrazine synthesis relied on the oxidative dimerization of an α‐amino ketone which was generated in situ from the corresponding α‐diketone via (regioselective) amination catalyzed by ω‐transaminases. The conversion of α‐diketones to pyrroles in the presence of β‐keto ester was achieved via Knorr pyrrole synthesis and the prevention of undesired dimerization of the α‐amino carbonyl intermediate was controlled by pH modification. In addition, a self‐sufficient approach for pyrrole synthesis was realized using a β‐amino ester as a substrate, which simultaneously served as amine donor. This latter approach exploited the reversible nature of biocatalytic amination to shuttle amine functionality across reactions partners with the reaction driven by irreversible pyrrole formation.

## Conflict of interest

The authors declare no conflict of interest.

## Supporting information

As a service to our authors and readers, this journal provides supporting information supplied by the authors. Such materials are peer reviewed and may be re‐organized for online delivery, but are not copy‐edited or typeset. Technical support issues arising from supporting information (other than missing files) should be addressed to the authors.

Supplementary
