# Supplementary material for: Chemo‐Enzymatic Synthesis of Pyrazines and Pyrroles
Source: Angew Chem Int Ed Engl. 2018 Nov 21;57(51):16760–3. doi: 10.1002/anie.201810555 (PMC6391939; doi:10.1002/anie.201810555)
Supplement: Supplementary file 1 — Supplementary [file ANIE-57-16760-s001.pdf]

Angewandte Chemie
Eine Zeitschrift der Gesellschaft Deutscher Chemiker

Supporting Information

# Chemo-Enzymatic Synthesis of Pyrazines and Pyrroles

*Jin Xu, Anthony P. Green,* and Nicholas J. Turner**

anie_201810555_sm_miscellaneous_information.pdf

## Table of Contents

Table of Contents ..... 2
1. General ..... 3
2. General procedure for analytical scale biotransformations ..... 4
2.1 General procedure for the chemo-enzymatic synthesis of pyrazines at analytical scale ..... 4
2.2 General procedure for the chemo-enzymatic synthesis of pyrroles at analytical scale ..... 4
2.3 Optimization for the chemo-enzymatic synthesis of pyrrole **2b** at analytic scale ..... 4
1. Preparation and characterization data for synthesized chemicals ..... 5
1,2,3,4,6,7,8,9-Octahydrophenazine (**4**): ..... 5
2,3,5,6-Tetramethylpyrazine (**5**): ..... 6
2,5-Diethyl-3,6-dimethylpyrazine (**6**): ..... 7
Ethyl 2,5-dimethyl-4-phenyl-1*H*-pyrrole-3-carboxylate (**7a**): ..... 8
Ethyl 2,5-dimethyl-4-(3'-(trifluoromethyl)phenyl)-1*H*-pyrrole-3-carboxylate (**8a**): ..... 9
Ethyl 2,5-dimethyl-4-(4'-(trifluoromethyl)phenyl)-1*H*-pyrrole-3-carboxylate (**9a**): ..... 10
Ethyl 2,5-dimethyl-4-(4'-chlorophenyl)-1*H*-pyrrole-3-carboxylate (**10a**): ..... 11
Ethyl 2,5-dimethyl-4-(4'-methoxyphenyl)-1*H*-pyrrole-3-carboxylate (**11a**): ..... 12
Ethyl 2,5-dimethyl-4-( 3',5'-dichlorophenyl)-1*H*-pyrrole-3-carboxylate (**12a**): ..... 13
Methyl 2,4,5-trimethyl-1*H*-pyrrole-3-carboxylate (**2b**): ..... 14
Methyl 2,5-dimethyl-4-phenyl-1*H*-pyrrole-3-carboxylate (**7b**): ..... 15
2. GC-FID analysis ..... 16
2.1 GC methods and conditions ..... 16
2.2 GC-FID traces ..... 18
3. GC-MS analysis ..... 25
3.1 GC-MS methods and conditions ..... 25
3.2 GC-MS traces ..... 27
4. NMR analysis ..... 30
4.1 NMR spectra of chemical standards and isolated chemicals ..... 30
5. Mass spectra ..... 42

## 1. General

All analytical grade chemicals and reagents were purchased from Sigma-Aldrich Company Ltd (Gillingham, UK) or fisher Scientific UK Ltd (Loughborough, UK), Acros organics, Alfa Aesar or Fluorochem UK Ltd (Hadfield, UK) and used without further purification. (*S*)-selective transaminase (ATA-113) and the (*R*)-selective transaminase (ATA-117) and cofactor (PLP) were generously provided by Codexis® (California, USA) as powdered lysate. Column chromatography was performed on silica gel (Fluka (Buchs, Switzerland), 220-440 mesh). TLC carried out on Polygram SIL G/UV254 and TLC plates were visualised under UV. Isolated products were analysed by $^{1}H$ NMR (400 MHz, $CDCl_3$) and $^{13}C$ NMR (100 MHz, $CDCl_3$) recorded on a Bruker™ Avance® 400 spectrometer. To determine reaction yields via NMR spectroscopy, 1,3-dinitrobenzene was added as internal standard. Chemical shifts were recorded as $\delta$ parts per million (ppm) referenced against residual solvent ($CHCl_3$) signals (7.26 ppm and 77.00 ppm for $^{1}H$ and $^{13}C$ respectively). $^{1}H$ multiplicities are reported as follows: singlet (s), doublet (d), triplet (t), quartet (q), pentet (p), dmultiplet (m), and broad resonance (br). High resolution mass spectrometry (HRMS) was performed on a Waters LCT-TOF instrument with data accurate to ± 0.001 Da.

## 2. General procedure for analytical scale biotransformations

### 2.1 General procedure for the chemo-enzymatic synthesis of pyrazines at analytical scale

To a solution of α-diketone (20 mM from a 800 mM stock in DMSO, 0.25 mL), (*S*)-aminotetralin (22 mM from a 880 mM stock in DMSO, 0.25 mL) and PLP·H2O (2.02 mM from 10.1 mM stock in 100 mM HEPES buffer, 1 mL) in HEPES buffer (100 mM, pH 7.4, 7.5 mL) was added a solution of commercially available ATA-113 (10 mg/mL, 1 mL). The pH of the reaction solution was adjusted to 7.4 prior to the addition of enzyme. The reaction mixture was incubated at 30 °C, 250 rpm for 72 hrs, basified to pH 12 by addition of 10 M NaOH and extracted with EtOAc (3 x 10 mL). The combined organics were dried over magnesium sulphate, filtered and concentrated under reduced pressure. The organic fractions were combined and dried over anhydrous $MgSO_4$ and analyzed by GC-MS or GC-FID with the previously purified standard. Details of columns and analytical methods, with chromatograms are in the Supplementary Information.

### 2.2 General procedure for the chemo-enzymatic synthesis of pyrroles at analytical scale

To a solution of α-diketone (20 mM from a 800 mM stock in DMSO, 0.25 mL), (*R*)-aminotetralin (22 mM from a 880 mM stock in DMSO, 0.25 mL), β-keto ester (200 mM from a 4 M stock in DMSO, 0.5 mL) and PLP·H2O (2.02 mM from 10.1 mM stock in 100 mM HEPES buffer, 1 mL) in HEPES buffer (100 mM, pH 7.4, 7 mL)was added a solution of commercially available ATA-117 (20 mg/mL, 1 mL). The pH of the reaction solution was adjusted to 7.4 prior to the addition of enzyme. The reaction mixture was incubated at 30 °C, 250 rpm for 72 hrs, basified to pH 12 by addition of 10 M NaOH and extracted with EtOAc (3 x 10 mL). The organic fractions were combined and dried over anhydrous $MgSO_4$ and analyzed by GC-MS or GC-FID with the previously purified standard. Details of columns and analytical methods, with chromatograms are in the Supplementary Information.

### 2.3 Optimization for the chemo-enzymatic synthesis of pyrrole 2b at analytic scale

The optimization for the chemo-enzymatic synthesis of pyrrole **2b** was carried out on four different pH (pH = 5, 6, 7.4, 9) and three different ratios of dialkyl α-diketone/β-keto ester (1:1, 1:3 and 1:10) via GC-MS analysis.

## 1. Preparation and characterization data for synthesized chemicals

**1,2,3,4,6,7,8,9-Octahydrophenazine (4):**

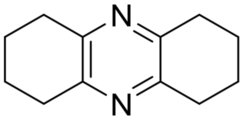


**Method A**: To a solution of α-diketone **1** (56 mg, 0.50 mmol), (*S*)-aminotetralin (83.5 mg, 0.55 mmol) and PLP·$H_2O$ (53 mg, 0.20 mmol) in HEPES buffer (100 mM, pH 7.4, 40 mL) and DMSO (5 mL) was added a solution of commercially available ATA-113 (10 mg/mL, 5 mL). The pH of the reaction solution was adjusted to 7.4 prior to the addition of enzyme. The reaction mixture was incubated at 30 °C, 250 rpm for 72 hrs, basified to pH 12 by addition of 10 M NaOH and extracted with EtOAc (3 x 50 mL). The combined organics were dried over magnesium sulphate, filtered and concentrated under reduced pressure. An aliquot (1 mL) of the organic extract was taken for analysis by GC before concentration. Preparative reverse phase HPLC ($H_2O$/MeOH = 50%:50%) followed by solvent evaporation afforded the pure title compound **4** (50 mg, 53%) as a white solid.

**Method B**: To a solution of α-diketone **1** (112 mg, 0.50 mmol), isopropylamine (300 mg, 5.08 mmol) and PLP·$H_2O$ (106 mg, 0.20 mmol) in HEPES buffer (100 mM, pH 7.4, 85 mL) was added a solution of commercially available ATA-113 (10 mg/mL, 10 mL) or ATA-117 (20 mg/mL,10 mL) and DMSO (5 mL). The pH of the reaction solution was adjusted to 7.4 prior to the addition of enzyme. The reaction mixture was incubated at 30 °C, 250 rpm for 72 hrs, basified to pH 12 by addition of 10 M NaOH and extracted with EtOAc (3 x 100 mL). The combined organics were dried over magnesium sulphate, filtered and concentrated under reduced pressure to afford the semi-purified product (**4**) which contained a minor impurity and DMSO. Flash chromatography of the remaining organics (DCM) afforded the pure title compound (**4**) as a white solid (61 mg, 65%).

**$^1$H NMR** (400 MHz, $CDCl_3$): $\delta_H$ 2.88 – 2.73 (8H, br.m, 1-C$H_2$ + 4-C$H_2$ + 6-C$H_2$ +9-C$H_2$), 1.92 – 1.74 (8H, br.m, 2-C$H_2$ + 3-C$H_2$ + 7-C$H_2$ + 8-C$H_2$). **$^{13}$C NMR** (101 MHz, $CDCl_3$): $\delta_C$ 149.3, 31.6, 22.8. **GC-MS**: m/z (EI) 188.2 (100%, $M^+$), 173.2 (8), 160.2 (32), 145.2 (5), 132.2 (10).

**2,3,5,6-Tetramethylpyrazine (5):**

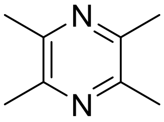

**Method A**: To a solution of α-diketone **2** (43 mg, 0.50 mmol), (*S*)-aminotetralin (83.5 mg, 0.55 mmol) and PLP·$H_2O$ (53 mg, 0.20 mmol) in HEPES buffer (100 mM, pH 7.4, 40 mL) and DMSO (5 mL) was added a solution of commercially available ATA-113 (10 mg/mL, 5 mL). The pH of the reaction solution was adjusted to 7.4 with 1 M HCl prior to the addition of enzyme. The reaction mixture was incubated at 30 °C, 250 rpm for 72 hrs, basified to pH 12 by addition of 10 M NaOH and extracted with EtOAc (3 x 50 mL). The combined organics were dried over magnesium sulphate, filtered and concentrated under reduced pressure. An aliquot (1 mL) of the organic extract was taken for analysis by GC before concentration. Flash chromatography of the remaining organics (Hexane/$Et_2O$ = 90%:10%) afforded the pure title compound (**5**) as a white solid (17 mg, 50%).

**Method B**: To a solution of α-diketone **2** (86 mg, 1.00 mmol), isopropylamine (300 mg, 5.08 mmol) and PLP·$H_2O$ (106 mg, 0.40 mmol) in HEPES buffer (100 mM, pH 7.4, 85 mL) and DMSO (5 mL) was added a solution of commercially available ATA-113 (10 mg/mL, 10 mL) or ATA-117 (20 mg/mL, 10 mL). The pH of the reaction solution was adjusted to 7.4 with 1 M HCl prior to the addition of enzyme. The reaction mixture was incubated at 30 °C, 250 rpm for 72 hrs, basified to pH 12 by addition of 10 M NaOH and extracted with EtOAc (3 x 100 mL). The combined organics were dried over magnesium sulphate, filtered and concentrated under reduced pressure to afford the semi-purified product (**5**) which contained a minor impurity and DMSO.

**$^1$H NMR** (400 MHz, $CDCl_3$): $\delta_H$ 2.43 – 2.47 (12H, s, 4 x C$H_3$). **$^{13}$C NMR** (101 MHz, $CDCl_3$): $\delta_C$ 148.1, 21.3. **GC-MS**: m/z (EI) 136.1 (100%, $M^+$), 121.2 (3), 94.2 (7), 54.0 (40), 42.1 (22).

**2,5-Diethyl-3,6-dimethylpyrazine (6):**

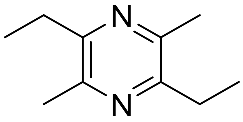

**Method A**: To a solution of α-diketone **3** (100 mg, 1.00 mmol), (*R*)-aminotetralin (167 mg, 1.10 mmol), and PLP·$H_2O$ (106 mg, 0.40 mmol) in HEPES buffer (100 mM, pH 7.4, 80 mL) and DMSO (10 mL) was added a solution of commercially available ATA-117 (40 mg/mL, 10 mL).The pH of the reaction solution was adjusted to 7.4 with 1 M HCl prior to the addition of enzyme. The reaction mixture was incubated at 30 °C, 250 rpm for 72 hrs, extracted with $Et_2O$ (3 x 100 mL) at pH 7.4. The combined organics were dried over magnesium sulphate, filtered and concentrated under reduced pressure. An aliquot (1 mL) of the organic extract was taken for analysis by GC before concentration. Flash chromatography of the remaining organics (Hexane/$Et_2O$ = 95%:5%) afforded the pure title compound (**6**) as a pale yellow solid (26 mg, 32%).

**$^1$H NMR** (400 MHz, $CDCl_3$): $\delta_H$ 2.75 (4H, q, *J* = 7.6 Hz, O$CH_2$$CH_3$), 2.49 (6H, s, 6'- $CH_3$ and 3'-$CH_3$), 1.24 (6H, t, *J* = 7.6 Hz, O$CH_2$$CH_3$).**$^{13}$C NMR** (101 MHz, $CDCl_3$): $\delta_C$ 152.7,147.8, 27.8, 20.9, 12.8. **GC-MS**: m/z (EI) 164.1 (74%, $M^+$), 149.1 (100), 136.1 (8), 121.1 (10).

**Ethyl 2,5-dimethyl-4-phenyl-1*H*-pyrrole-3-carboxylate (7a):**

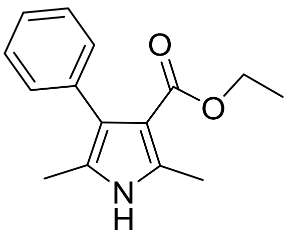

**Method A**: To a solution of α-diketone **7** (75 mg, 0.50 mmol), (*R*)-aminotetralin (83.5 mg, 0.55 mmol), β-keto ester **a** (650 mg, 5.00 mmol) and PLP·$H_2O$ (53 mg, 0.20 mmol) in HEPES buffer (100 mM, pH 7.4, 85 mL) and DMSO (5 mL) was added a solution of commercially available ATA-117 (20 mg/mL, 10 mL). The pH of the reaction solution was adjusted to 7.4 with 1 M HCl prior to the addition of enzyme. The reaction mixture was incubated at 30 °C, 250 rpm for 72 hrs, acidified to pH 4 by addition of 3 M HCl and extracted with EtOAc (3 x 100 mL). The combined organics were dried over magnesium sulphate, filtered and concentrated under reduced pressure. An aliquot (1 mL) of the organic extract was taken for analysis by GC before concentration. Flash chromatography of the remaining organics (Hexane/EtOAc = 95%:5% then Hexane/EtOAc = 90%:10%) afforded the semi-purified product (**7a**) which contained a minor impurity. A second round of flash chromatography (DCM) afforded the pure title compound (**7a**) as a pale yellow solid (45 mg, 38%).

**Method B**: To a solution of α-diketone **7** (75 mg, 0.50 mmol), racemic β-amino ester **13** (650 mg, 2.50 mmol of (*R*)-enantiomer) and PLP·$H_2O$ (53 mg, 0.20 mmol) in HEPES buffer (100 mM, pH 7.4, 85 mL) and DMSO (5 mL) was added a solution of commercially available ATA-117 (20 mg/mL, 10 mL). The pH of the reaction solution was adjusted to 7.4 with 1 M HCl prior to the addition of enzyme. The reaction mixture was incubated at 30 °C, 250 rpm for 72 hrs, acidified to pH 4 by addition of 3 M HCl and extracted with EtOAc (3 x 100 mL). Product **7a** was identified by GC-FID analysis by comparison with the previously purified standard.

**HRMS**: (Found $[M+H]^+$, 244.1328. $C_{15}H_{18}NO_2$, requires *M*, 244.1332). **IR** ($\upsilon_{max}/cm^{-1}$): 3304, 2979, 1664, 1496, 1445, 1329, 1288, 1170, 1090,784, 756, 700. **$^1$H NMR** (400 MHz, $CDCl_3$): $\delta_H$ 8.15 (1H, br.s, N*H*), 7.38 – 7.28 (2H, m, Ar-*H*), 7.26 – 7.21 (3H, m, Ar-*H*), 4.09 (2H, q, *J* = 7.1 Hz, O$CH_2$$CH_3$), 2.50 and 2.10 (2 x 3H, s, 5'- $CH_3$ and 2'-$CH_3$), 1.06 (3H, t, *J* = 7.1 Hz, O$CH_2$$CH_3$). **$^{13}$C NMR** (101 MHz, $CDCl_3$): $\delta_C$ 165.8, 136.2, 133.8, 130.4, 127.3, 125.8, 123.4, 122.4, 110.6, 59.0, 14.0, 13.6, 11.2. **GC-MS**: m/z (EI) 243.1 (91%, $M^+$), 214.1 (100), 198.1 (30), 168.1 (25), 128 (13).

**Ethyl 2,5-dimethyl-4-(3'-(trifluoromethyl)phenyl)-1*H*-pyrrole-3-carboxylate (8a):**

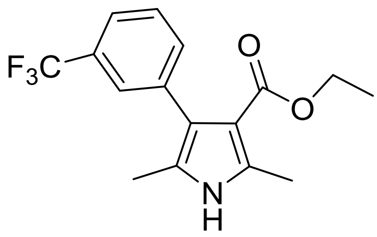

To a solution of diketone **8** (114 mg, 0.50 mmol), (*R*)-aminotetralin (83.5 mg, 0.55 mmol), β-keto ester **a** (650 mg, 5.00 mmol) and PLP·$H_2O$ (53 mg, 0.20 mmol) in HEPES buffer (100 mM, pH 7.4, 80 mL) and DMSO (10 mL) was added a solution of commercially available ATA-117 (20 mg/mL, 10 mL). The pH of the reaction solution was adjusted to 7.4 with 1 M HCl prior to the addition of enzyme. The reaction mixture was incubated at 30 °C, 250 rpm for 72 hrs acidified to pH 4 by addition of 3 M HCl and extracted with EtOAc (3 x 100 mL). The combined organics were dried over magnesium sulphate, filtered and concentrated under reduced pressure. An aliquot (1 mL) of the organic extract was taken for analysis by GC before concentration. Flash chromatography of the remaining organics (Hexane/EtOAc = 95%:5% then Hexane/EtOAc = 90%:10%) afforded the semi-purified product (**8a**) which contained a minor impurity. A second round of flash chromatography (DCM) afforded the pure title compound (**8a**) as a pale yellow solid (53 mg, 34%).

**HRMS**: (Found $[M+H]^+$, 312.1206. $C_{16}H_{17}F_3NO_2$, requires *M*, 312.1206). **IR** ($\upsilon_{max}$ /$cm^{-1}$): 3305, 2980, 2356, 2337, 1664, 1423, 1325, 1286, 1270, 1179, 1163, 1124, 1099, 1073. **$^1$H NMR** (400 MHz, $CDCl_3$): $\delta_H$ 8.08 (1H, br.s, N*H*), 7.54 – 7.39 (4H, m, Ar-*H*), 4.06 (2H, q, *J* = 7.1 Hz, O$CH_2$$CH_3$), 2.53 and 2.11 (2 x 3H, s, 5'- $CH_3$ and 2'-$CH_3$), 1.02 (3H, t, *J* = 7.1 Hz, O$CH_2$$CH_3$). **$^{13}$C NMR** (101 MHz, $CDCl_3$): $\delta_C$ 165.5, 137.1, 134.5, 133.7, 129.7 (q, $^2J$(C,F) = 32 Hz), 127.7, 127.3 (q, $^3J$(C,F) = 4.0 Hz), 124.1 (q, *J*(C,F) = 271 Hz, $CF_3$), 123.9, 122.6 (q, $^3J$(C,F) = 4.0 Hz), 121.1, 110.6, 59.2, 13.9, 13.6, 11.2. **GC-MS**: m/z (EI) 311.1 (100%, $M^+$), 282.1 (96), 266.1 (30), 236.1(13), 168.1 (16).

**Ethyl 2,5-dimethyl-4-(4'-(trifluoromethyl)phenyl)-1*H*-pyrrole-3-carboxylate (9a):**

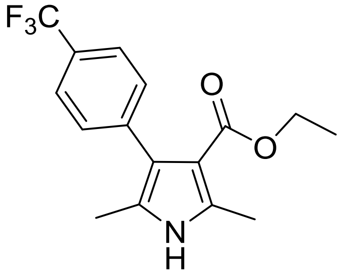

To a solution of diketone **9** (114 mg, 0.50 mmol), (*R*)-aminotetralin (83.5 mg, 0.55 mmol), β-keto ester **a** (650 mg, 5.00 mmol) and PLP·$H_2O$ (53 mg, 0.20 mmol) in HEPES buffer (100 mM, pH 7.4, 80 mL) and DMSO (10 mL) was added a solution of commercially available ATA-117 (20 mg/mL, 10 mL). The pH of the reaction solution was adjusted to 7.4 with 1 M HCl prior to the addition of enzyme. The reaction mixture was incubated at 30 °C, 250 rpm for 72 hrs, acidified to pH 4 by addition of 3 M HCl and extracted with EtOAc (3 x 100 mL). The combined organics were dried over magnesium sulphate, filtered and concentrated under reduced pressure. An aliquot (1 mL) of the organic extract was taken for analysis by GC before concentration. Flash chromatography of the remaining organics (Hexane/EtOAc = 95%:5% then Hexane/EtOAc = 90%:10%) afforded the semi-purified product (**9a**) which contained a minor impurity. A second round of flash chromatography (DCM) afforded the pure title compound (**9a**) as a pale yellow solid (32 mg, 21%).

**HRMS**: (Found $[M+H]^+$, 312.1205. $C_{16}H_{17}F_3NO_2$, requires *M*, 312.1206). **IR** ($\upsilon_{max}$ /$cm^{-1}$): 3304, 2981, 1668, 1387, 1321, 1294, 1161, 1120, 1094, 1067. **$^1$H N MR** (400 MHz, $CDCl_3$): $\delta_H$ 8.08 (1H, br.s, N*H*), 7.54 – 7.39 (2H, m, Ar-*H*), 7.39 – 7.30 (3H, m, Ar-*H*), 4.09 (2H, q, *J* = 7.1 Hz, O$CH_2$CH$_3$), 2.52 and 2.11 (2 x 3H, s, 5'- $CH_3$ and 2'-$CH_3$), 1.07 (3H, t, *J* = 7.1 Hz, $OCH_2CH_3$). **$^{13}$C NMR** (101 MHz, $CDCl_3$): $\delta_C$ 165.4, 140.1, 134.3, 130.6, 127.9 (q, $^2J$(C,F) = 32 Hz), 124.5 (q, *J*(C,F) = 270 Hz, $CF_3$), 124.2 (q, $^3J$(C,F) = 4.0 Hz), 123.2, 121.3, 110.5, 59.2, 14.0, 13.7, 11.1. **GC-MS**: m/z (EI): 311.1 (77%, $M^+$), 282.1 (100), 266.1 (30), 236.1(8), 168.1 (11).

**Ethyl 2,5-dimethyl-4-(4'-chlorophenyl)-1*H*-pyrrole-3-carboxylate (10a):**

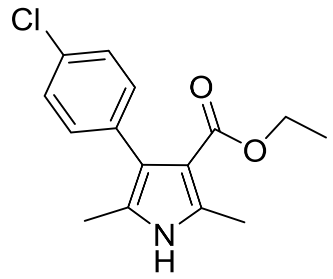

To a solution of diketone **10** (96 mg, 0.50 mmol), (*R*)-aminotetralin (83.5 mg, 0.55 mmol), β-keto ester **a** (650 mg, 5.00 mmol) and PLP·$H_2O$ (53 mg, 0.20 mmol) in HEPES buffer (100 mM, pH 7.4, 80 mL) and DMSO (10 mL) was added a solution of commercially available ATA-117 (20 mg/mL, 10 mL). The pH of the reaction solution was adjusted to 7.4 with 1 M HCl prior to the addition of enzyme. The reaction mixture was incubated at 30 °C, 250 rpm for 72 hrs, acidified to pH 4 by addition of 3 M HCl and extracted with EtOAc (3 x 100 mL). The combined organics were dried over magnesium sulphate, filtered and concentrated under reduced pressure. An aliquot (1 mL) of the organic extract was taken for analysis by GC before concentration. Flash chromatography of the remaining organics (Hexane/EtOAc = 95%:5% then Hexane/EtOAc = 90%:10%) afforded the semi-purified product (**10a**) which contained a minor impurity. A second round of flash chromatography (DCM) afforded the pure title compound (**10a**) as a pale yellow solid (50 mg, 36%).

**HRMS**: (Found $[M+H]^+$, 278.0948. $C_{15}H_{17}ClNO_2$, requires *M*, 278.0942). **IR** ($\upsilon_{max}$ /$cm^{-1}$): 3299, 2979, 1666, 1491, 1433, 1287, 1167, 1088, 1015, 828, 783. **$^1$H NMR** (400 MHz, $CDCl_3$): $\delta_H$ 8.08 (1H, br.s, N*H*), 7.36 – 7.23 (2H, m, Ar-H), 7.21 – 7.14 (3H, m, Ar-*H*), 4.10 (2H, q, *J* = 7.1 Hz, O$CH_2$$CH_3$), 2.49 and 2.08 (2 x 3H, s, 5'- $CH_3$ and 2'-$CH_3$), 1.11 (3H, t, *J* = 7.1 Hz, $OCH_2CH_3$). **$^{13}$C NMR** (101 MHz, $CDCl_3$): $\delta_C$ 165.6, 134.7, 134.1, 131.7, 127.5, 123.6, 121.3, 110.4, 59.1, 14.1, 13.7, 11.1. **GC-MS**: m/z (EI): 277.1 (80%, $M^+$), 248.1 (100), 232.1 (25), 207.1(12), 168.1 (30).

**Ethyl 2,5-dimethyl-4-(4'-methoxyphenyl)-1*H*-pyrrole-3-carboxylate (11a):**

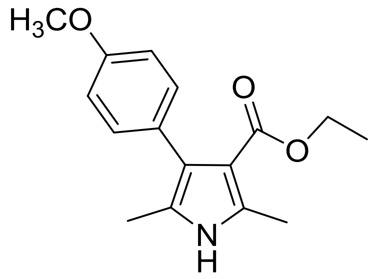


To a solution of diketone **11** (94 mg, 0.50 mmol), (*R*)-aminotetralin (83.5 mg, 0.55 mmol), β-keto ester **a** (650 mg, 5.00 mmol) and PLP·$H_2O$ (53 mg, 0.20 mmol) in HEPES buffer (100 mM, pH 7.4, 80 mL) and DMSO (10 mL) was added a solution of commercially available ATA-117 (20 mg/mL, 10 mL). The pH of the reaction solution was adjusted to 7.4 with 1 M HCl prior to the addition of enzyme. The reaction mixture was incubated at 30 °C, 250 rpm for 72 hrs, acidified to pH 4 by addition of 3 M HCl and extracted with EtOAc (3 x 100 mL). The combined organics were dried over magnesium sulphate, filtered and concentrated under reduced pressure. An aliquot (1 mL) of the organic extract was taken for analysis by GC before concentration. Flash chromatography of the remaining organics (Hexane/EtOAc = 95%:5% then Hexane/EtOAc = 90%:10%) afforded the semi-purified product (**11a**) which contained a minor impurity. A second round of flash chromatography (DCM) afforded the pure title compound (**11a**) as a pale red solid (40 mg, 31%).

**HRMS**: (Found $[M+H]^+$, 274.1442. $C_{15}H_{17}ClNO_2$, requires *M*, 274.1438). **IR** ($\upsilon_{max}$ /$cm^{-1}$): 3306, 2979, 2929, 1666, 1535, 1506, 1439, 1287, 1241, 1161, 1089, 1039, 830, 808, 786. **$^1$H NMR** (400 MHz, $CDCl_3$): $\delta_H$ 7.98 (1H, br.s, N*H*), 7.21 – 7.10 (2H, m, Ar-*H*), 6.98 – 6.77 (3H, m, Ar-*H*), 4.10 (2H, q, *J* = 7.1 Hz, O$CH_2$$CH_3$), 3.82 (3H, s, O$CH_3$), 2.50 and 2.10 (2 x 3H, s, 5'- $CH_3$ and 2'-$CH_3$), 1.10 (3H, t, *J* = 7.1 Hz, O$CH_2CH_3$). **$^{13}$C NMR** (101 MHz, $CDCl_3$): $\delta_C$ 165.8, 157.9, 133.7, 131.4, 128.5, 123.3, 122.1, 112.8, 110.7, 59.0, 55.2, 14.1, 13.8, 11.2. **GC-MS**: m/z (EI): 273.1 (100%, $M^+$), 244.1 (79), 228.1 (18), 200.1(6), 168.1 (10).

**Ethyl 2,5-dimethyl-4-( 3',5'-dichlorophenyl)-1*H*-pyrrole-3-carboxylate (12a):**

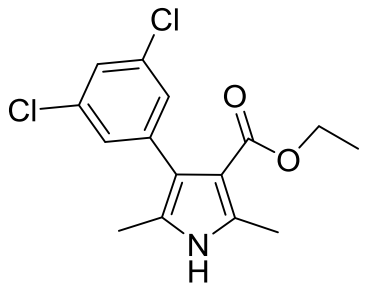


To a solution of diketone **12 (**57 mg, 0.25 mmol), (*R*)-aminotetralin (42 mg, 0.28 mmol), β-keto ester **a** (325 mg, 2.50 mmol) and PLP·$H_2O$ (53 mg, 0.20 mmol) in HEPES buffer (100 mM, pH 7.4, 80 mL) and DMSO (10 mL) was added a solution of commercially available ATA-117 (20 mg/mL, 10 mL). The pH of the reaction solution was adjusted to 7.4 with 1 M HCl prior to the addition of enzyme. The reaction mixture was incubated at 30 °C, 250 rpm for 72 hrs, acidified to pH 4 by addition of 3 M HCl and extracted with EtOAc (3 x 100 mL). The combined organics were dried over magnesium sulphate, filtered and concentrated under reduced pressure. An aliquot (1 mL) of the organic extract was taken for analysis by GC before concentration. Flash chromatography of the remaining organics (Hexane/EtOAc = 95%:5% then Hexane/EtOAc = 90%:10%) afforded the semi-purified product (**12a**) which contained a minor impurity. A second round of flash chromatography (DCM) afforded the pure title compound (**12a**) as a grey solid (52 mg, 65%).

**HRMS**: (Found $[M+Na]^+$, 344.0371. $C_{15}H_{15}{}^{35}Cl_2NO_2Na$, requires *M*, 344.0371). **IR** ($\upsilon_{max}$ /$cm^{-1}$): 3303, 2979, 2926, 1668, 1586, 1556, 1438, 1288, 1175, 1096, 800, 782. **$^1$H NMR** (400 MHz, $CDCl_3$): $\delta_H$ 8.14 (1H, br.s, N*H*), 7.25 – 7.22 (2H, m, Ar-*H*), 7.17 – 7.09 (3H, m, Ar-*H*), 4.10 (2H, q, *J* = 7.1 Hz, O$CH_2$$CH_3$), 2.50 and 2.11 (2 x 3H, s, 5'- $CH_3$ and 2'-$CH_3$), 1.11 (3H, t, *J* = 7.1 Hz, O$CH_2$$CH_3$). **$^{13}$C NMR** (101 MHz, $CDCl_3$): $\delta_C$ 165.3, 139.4, 134.7, 133.6, 129.0, 125.9, 124.1, 120.0, 110.4, 59.3, 14.0, 13.6, 11.2. **GC-MS**: m/z (EI): 315.2(10%), 313.1(43), 311.0 (80, $M^+$), 282.0 (100), 266.0 (27).

**Methyl 2,4,5-trimethyl-1*H*-pyrrole-3-carboxylate (2b):**

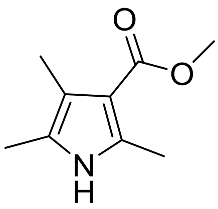

To a solution of α-diketone **7** (86 mg, 1.00 mmol), (*R*)-aminotetralin (167 mg, 1.10 mmol), β-keto ester **b** (1160 mg, 10.00 mmol) and PLP·$H_2O$ (106 mg, 0.40 mmol) in HEPES buffer (100 mM, pH 7.4, 80 mL) and DMSO (10 mL) was added a solution of commercially available ATA-117 (40 mg/mL, 10 mL). The pH of the reaction solution was adjusted to 7.4 with 1 M HCl prior to the addition of enzyme. The reaction mixture was incubated at 30 °C, 250 rpm for 72 hrs, extracted with $Et_2O$ (3 x 100 mL) at pH 7.4. The combined organics were dried over magnesium sulphate, filtered and concentrated under reduced pressure. An aliquot (1 mL) of the organic extract was taken for analysis by GC before concentration. Flash chromatography of the remaining organics (Hexane/$Et_2O$ = 95%:5%) afforded the semi-purified product (**2b**) which contained a minor impurity. A second round of flash chromatography (DCM) afforded the pure title compound (**2b**) as a pale yellow solid (49.6 mg, 30%).

**HRMS**: (Found $[M+H]^+$, 168.0993. $C_9H_{14}NO_2$, requires *M*, 168.1019). **$^1$H NMR** (400 MHz, $CDCl_3$): $\delta_H$ 7.96 (1H, br.s, N*H*), 3.61 (3H, s, O*CH$_3$*), 2.44, 2.14 and 2.11 (3 x 3H, s, 5'- *CH$_3$*, 4'- *CH$_3$* and 2'-*CH$_3$*). **$^{13}$C NMR** (101 MHz, $CDCl_3$): $\delta_C$ $^{13}$C NMR (101 MHz, Chloroform-*d*) δ 166.9, 133.7, 122.0, 115.9, 110.5, 50.3, 13.8, 10.9, 10.4.

**Methyl 2,5-dimethyl-4-phenyl-1*H*-pyrrole-3-carboxylate (7b):**

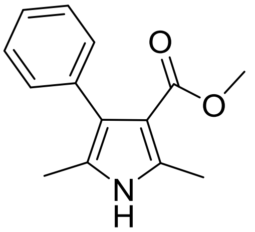


To a solution of α-diketone **7** (75 mg, 0.50 mmol), (*R*)-aminotetralin (83.5 mg, 0.55 mmol), β-keto ester **b** (580 mg, 5.00 mmol) and PLP·$H_2O$ (53 mg, 0.20 mmol) in HEPES buffer (100 mM, pH 7.4, 85 mL) and DMSO (5 mL) was added a solution of commercially available ATA-117 (20 mg/mL, 10 mL). The pH of the reaction solution was adjusted to 7.4 with 1 M HCl prior to the addition of enzyme. The reaction mixture was incubated at 30 °C, 250 rpm for 72 hrs, acidified to pH 4 by addition of 3 M HCl and extracted with EtOAc (3 x 100 mL). The combined organics were dried over magnesium sulphate, filtered and concentrated under reduced pressure. An aliquot (1 mL) of the organic extract was taken for analysis by GC before concentration. Flash chromatography of the remaining organics (Hexane/EtOAc = 95%:5% then Hexane/EtOAc = 90%:10%) afforded the semi-purified product (**7b**) which contained a minor impurity. A second round of flash chromatography (DCM) afforded the pure title compound (**7b**) as a pale yellow solid (47.8 mg, 42%).

**HRMS**: (Found $[M+H]^+$, 230.1176. $C_{14}H_{16}NO_2$, requires *M*, 230.1176). **IR** ($\upsilon_{max}/cm^{-1}$): 3306, 2946, 1667, 1496, 1447, 1334, 1291, 1190, 1165, 1091,787, 757, 701. **$^1$H NMR** (400 MHz, $CDCl_3$): $\delta_H$ 8.00 (1H, br.s, N*H*), 7.38 – 7.30 (2H, m, Ar-*H*), 7.24-7.21 (3H, m, Ar-*H*), 3.61 (3H, s, O$CH_3$), 2.51 and 2.12 (2 x 3H, s, 5'- $CH_3$ and 2'-$CH_3$). **$^{13}$C NMR** (101 MHz, $CDCl_3$): $\delta_C$ 166.2, 136.0, 133.9, 130.3, 127.4, 125.9, 123.5, 122.5, 110.3, 50.4, 13.7, 11.2. **GC-MS**: m/z (EI) 229.1 (100%, $M^+$), 214.1 (39), 198 (41), 168.1 (34), 128 (11).

## 2. GC-FID analysis

### 2.1 GC methods and conditions

**GC method A**: GC analysis was performed on an Agilent™ 6850 gas chromatograph equipped with a FID detector. Standards were dissolved in EtOAc and biotransformations were extracted with EtOAc. 10 µL of sample was injected onto an Agilent J&W HP-1 (30 mm x 0.32 mm i.d. x 0.25 µm film thickness) column at 200 °C. The oven temperature was set at 50 °C for 1 min and raised at 2 °C/min to 120 °C and held for 1 min, then at 10 °C/min to 320 °C. The carrier gas flow rate was 1.3 mL/min. The following gas chromatograph parameters were used: detector temperature was set at 250 °C, Split ratio 10:1, pressure 6.8 psi and velocity 24 cm/sec.

**GC method B**: GC analysis was performed on an Agilent™ 6850 gas chromatograph equipped with a FID detector. Standards were dissolved in EtOAc and biotransformations were extracted with EtOAc. 10 µL of sample was injected onto an Agilent J&W HP-1 (30 mm x 0.32 mm i.d. x 0.25 µm film thickness) column at 200 °C. The oven temperature was set at 50 °C for 1 min and raised at 5 °C/min to 100 °C, then at 2 °C/min to 150 °C and held for 1 min, then at 10 °C/min to 280 °C. The carrier gas flow rate was 1.3 mL/min. The following gas chromatograph parameters were used: detector temperature was set at 250 °C, Split ratio 10:1, pressure 6.8 psi and velocity 24 cm/sec.

**Table S1.** Retention time table for GC-FID analysis

| Compound | GC Method | Retention time (min) |
|---|---|---|
| **7** | GC Method A | 20.0 |
| | GC Method B | 12.9 |
| **8** | GC Method A | 19.3 |
| **9** | GC Method A | 18.7 |
| **10** | GC Method A | 29.2 |
| **11** | GC Method A | 37.2 |
| **12** | GC Method A | 36.8 |
| **a** | GC Method A | 8.9 |
| | GC Method B | 7.3 |
| **b** | GC Method B | 5.6 |
| **7a** | GC Method A | 47.3 |
| | GC Method B | 43.8 |
| **8a** | GC Method A | 47.2 |
| **9a** | GC Method A | 47.3 |
| **10a** | GC Method A | 49.2 |
| **11a** | GC Method A | 49.5 |
| **12a** | GC Method A | 50.4 |
| **7b** | GC Method A | 43.0 |
| **aminotetralin** | GC Method A | 31.1 |
| | GC Method B | 20 |
| **Tetralone** | GC Method A | 31.9 |
| | GC Method B | 20.6 |
| **1,3-dinitrobenzene** | GC Method A | 34.6 |

### 2.2 GC-FID traces

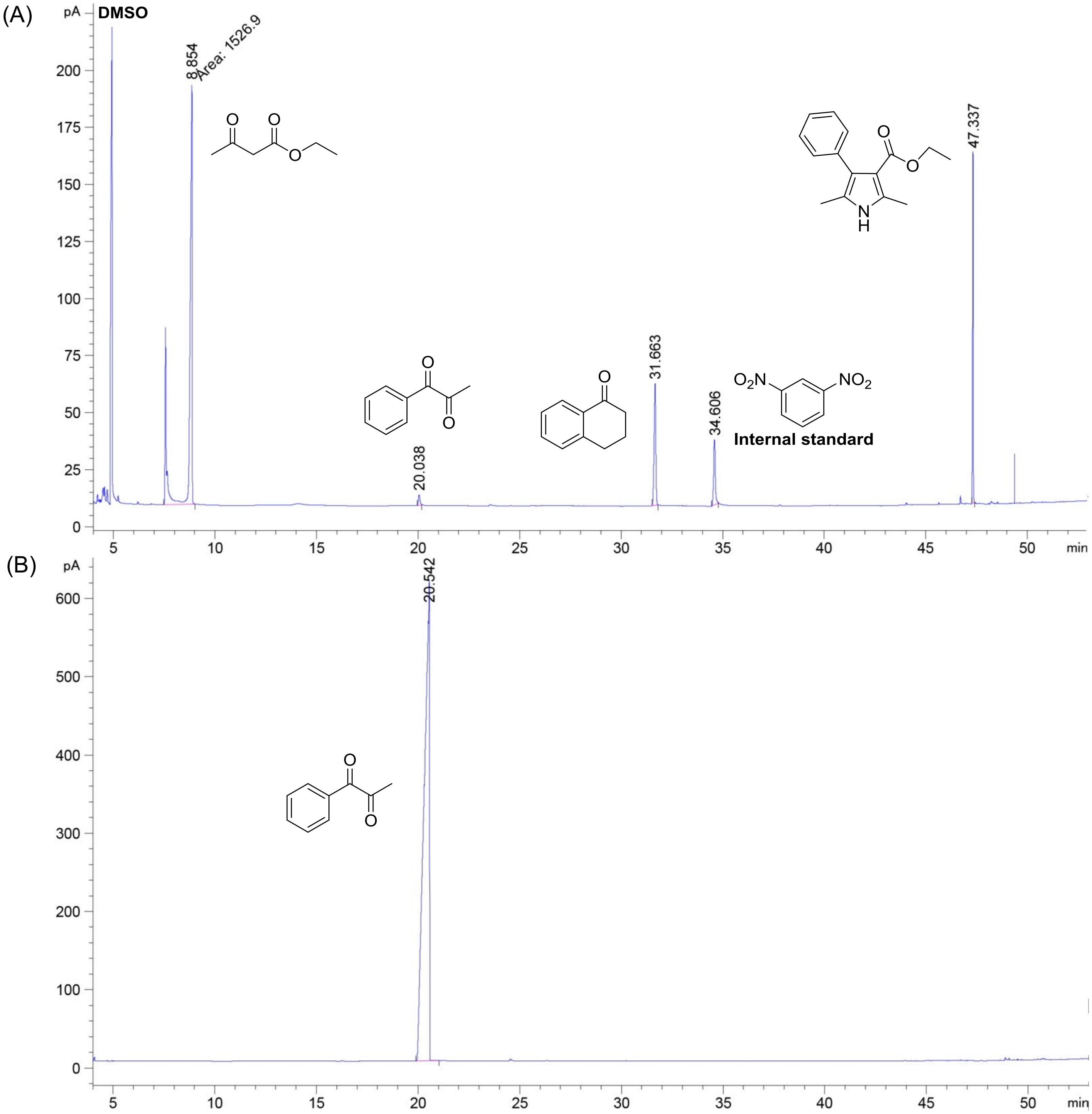


**Figure S1**: GC-FID traces: (A) Conversion of aryl α-diketone **7** to pyrrole **7b** in the presence of β-keto ester **b** and (B) α-diketone **7** standard.

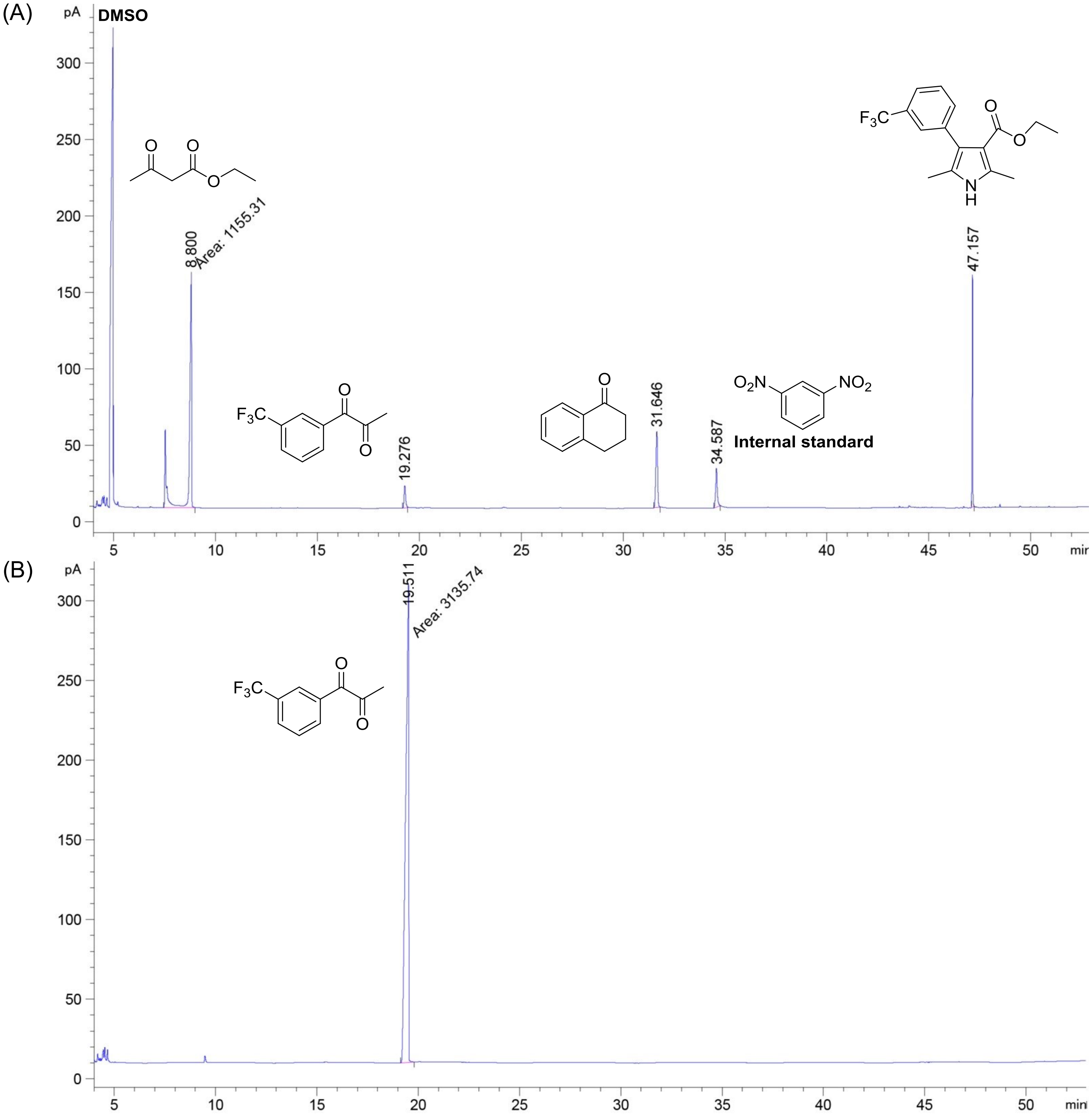


**Figure S2**: GC-FID traces: (A) Conversion of aryl α-diketone **8** to pyrrole **8b** in the presence of β-keto ester **b** and (B) α-diketone **8** standard.

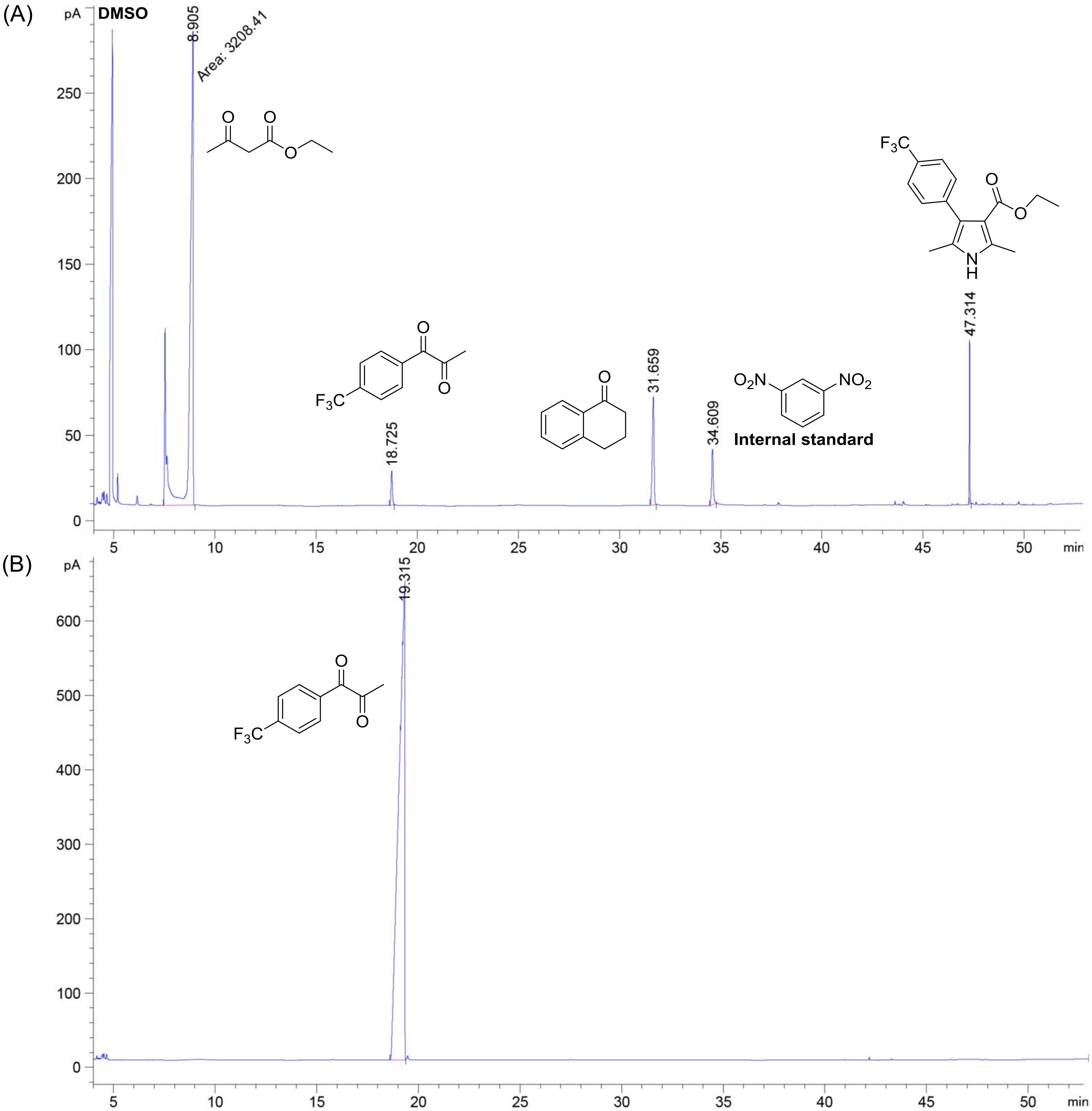


**Figure S3**: GC-FID traces: (A) Conversion of aryl α-diketone **9** to pyrrole **9b** in the presence of β-keto ester **b** and (B) α-diketone **9** standard.

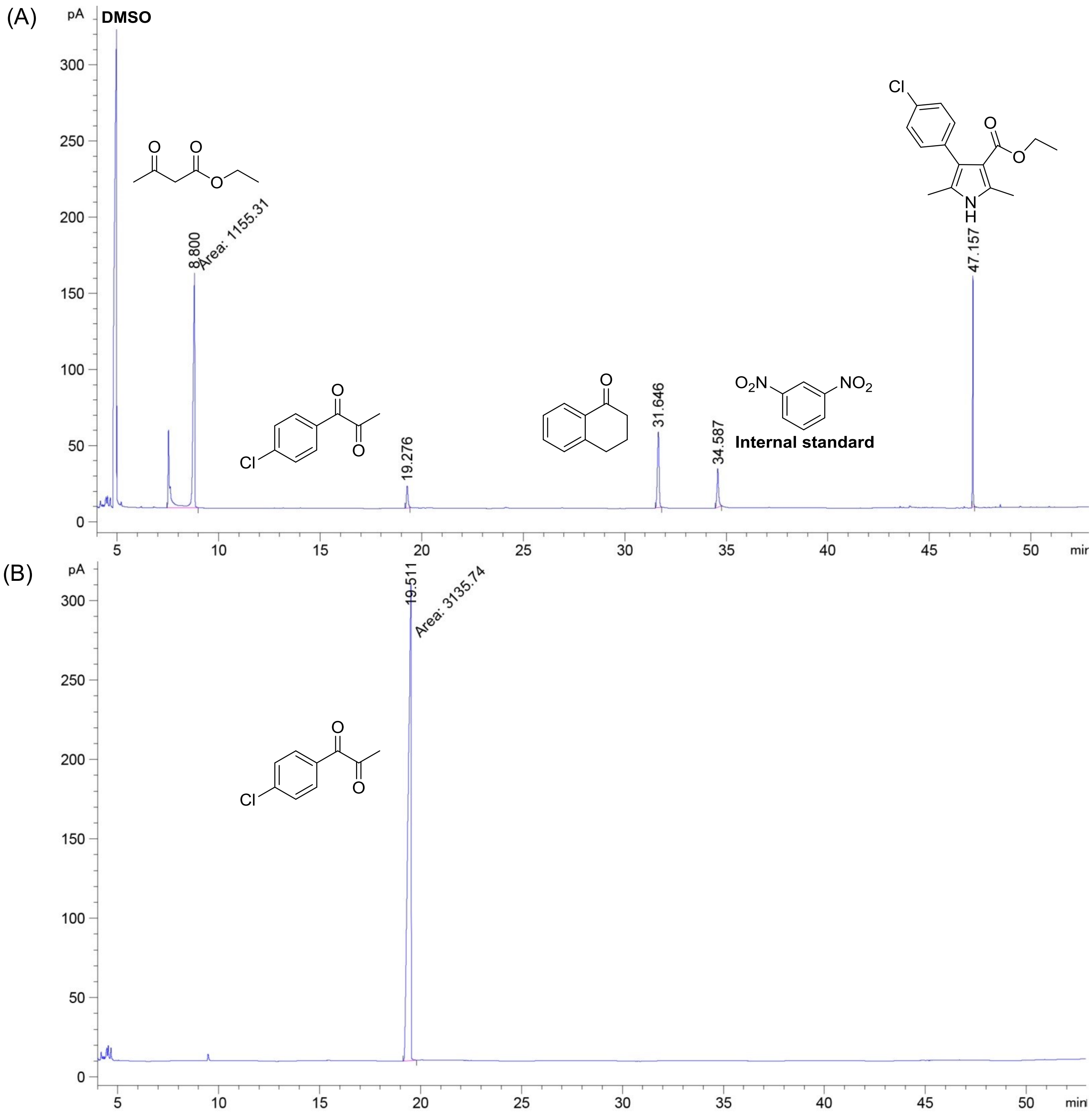


**Figure S4**: GC-FID traces: (A) Conversion of aryl α-diketone **10** to pyrrole **10b** in the presence of β-keto ester **b** and (B) α-diketone **10** standard.

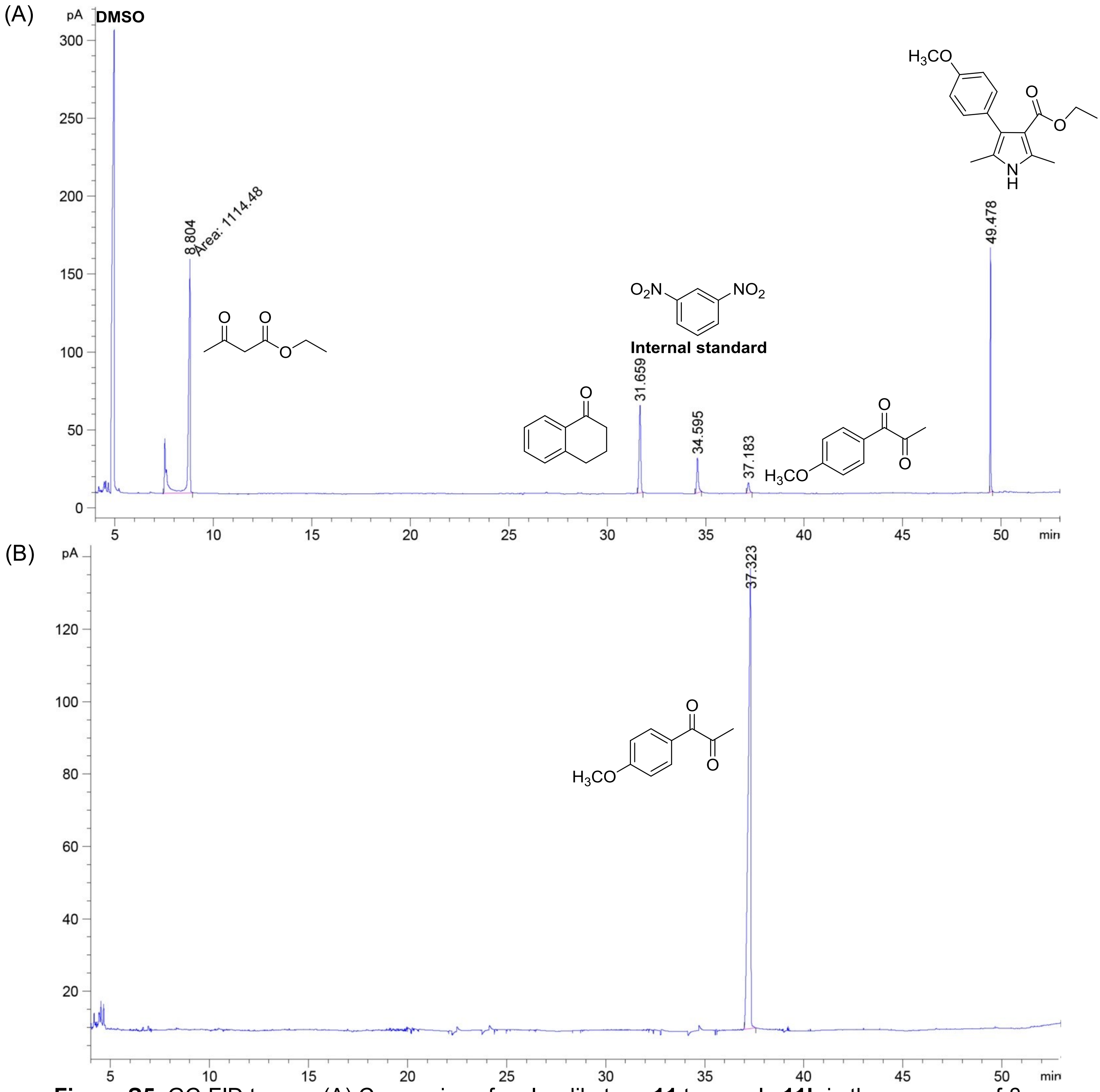


**Figure S5**: GC-FID traces: (A) Conversion of aryl α-diketone **11** to pyrrole **11b** in the presence of β-keto ester **b** and (B) α-diketone **11** standard.

(A)

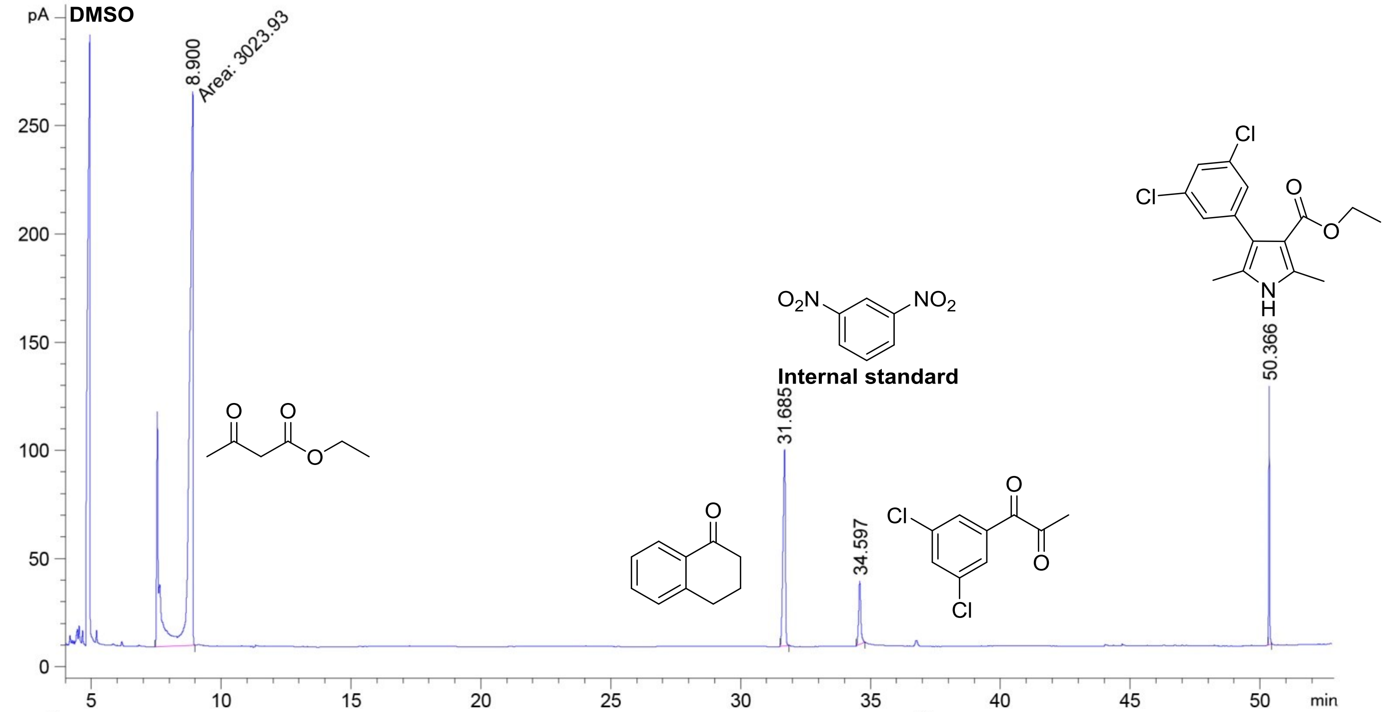


(B)

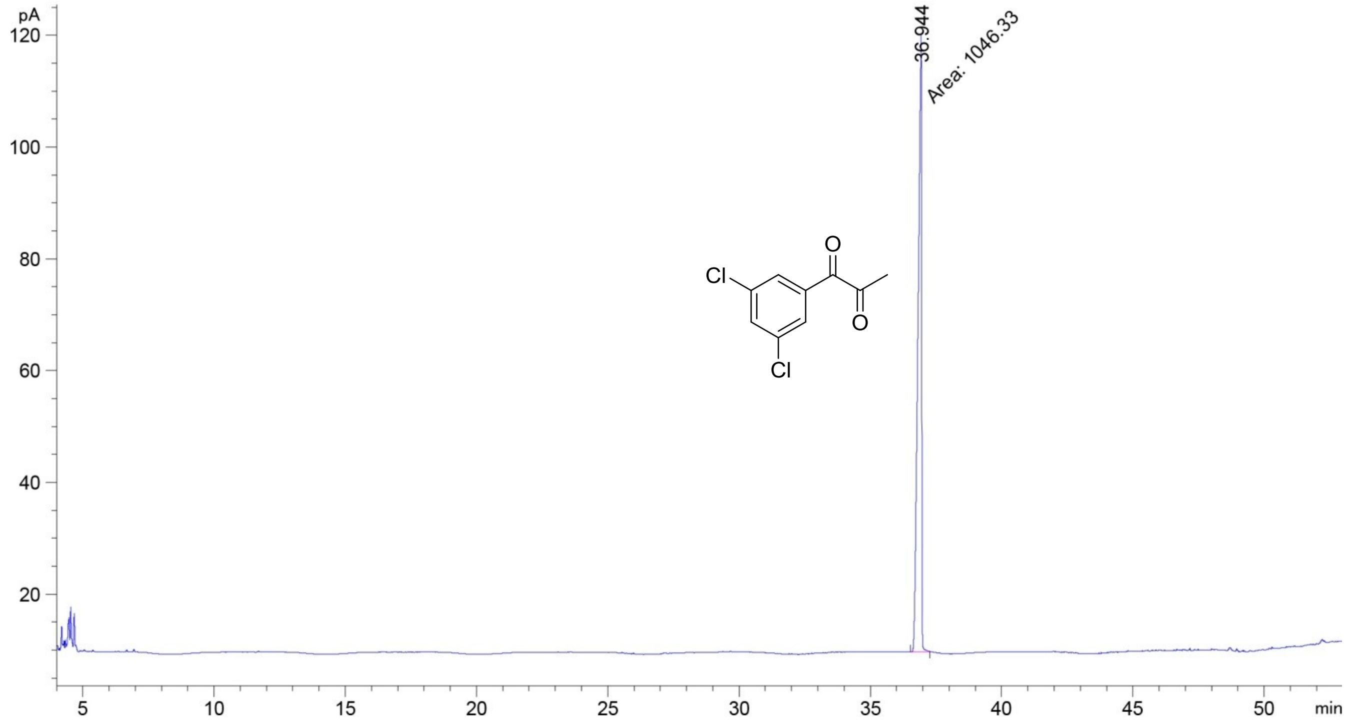


**Figure S6**: GC-FID traces: (A) Conversion of aryl α-diketone **12** to pyrrole **12b** in the presence of β-keto ester **b** and (B) α-diketone **12** standard.

(A)

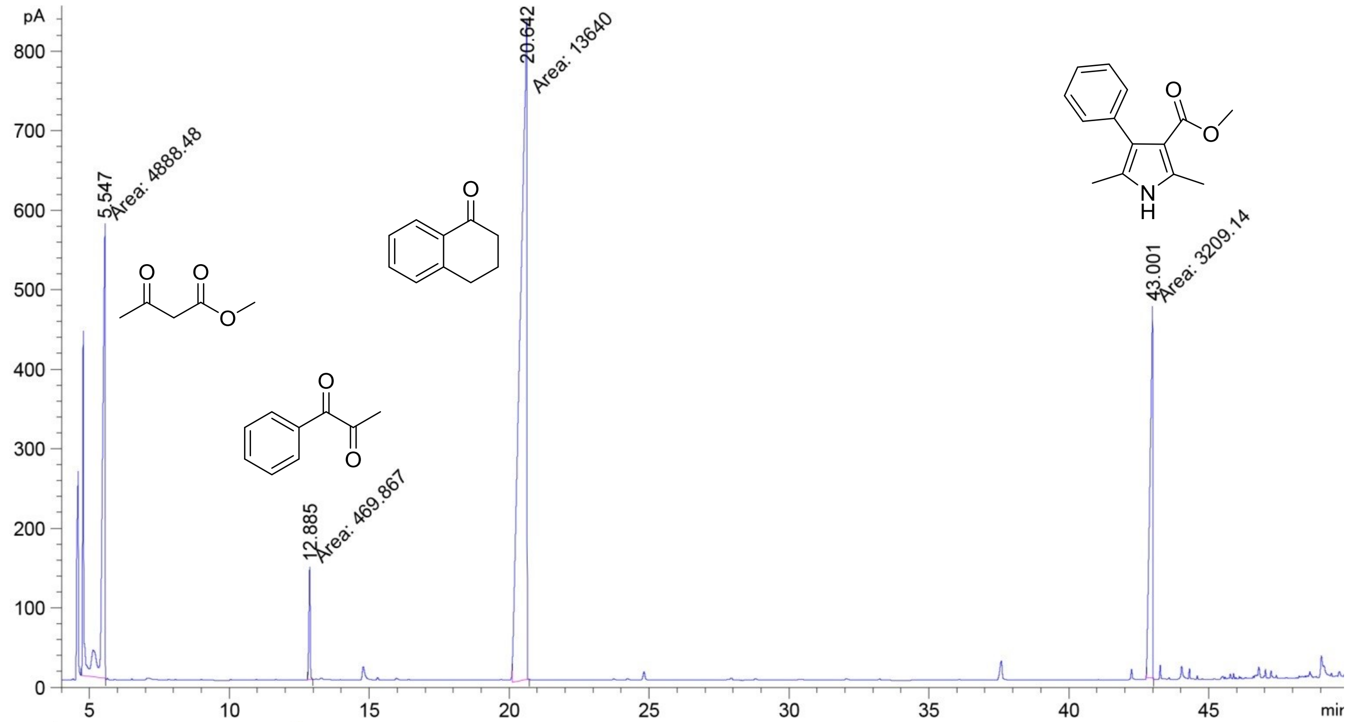


(B)

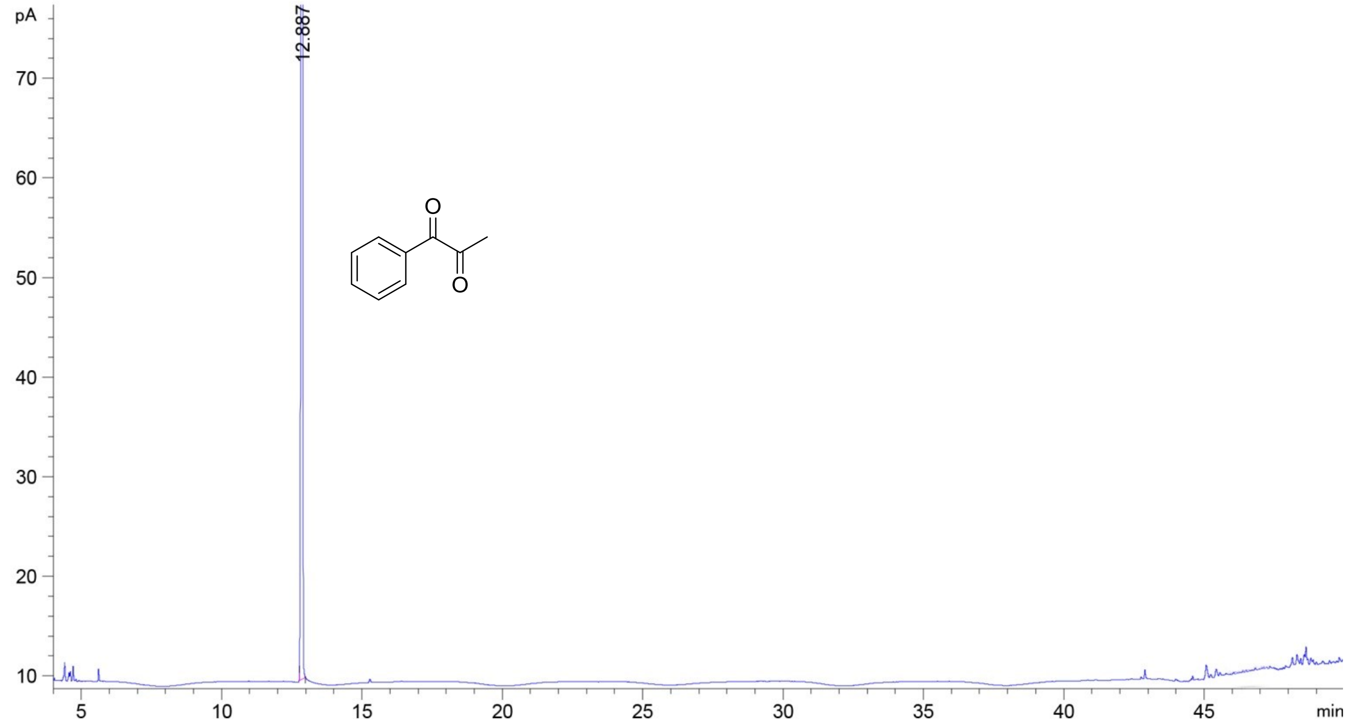


**Figure S7**: GC-FID traces: (A) Conversion of aryl α-diketone **7** to pyrrole **7a** in the presence of β-keto ester **a** and (B) α-diketone **7** standard

# 3. GC-MS analysis

## 3.1 GC-MS methods and conditions

**GC-MS method A**: GC-MS analysis were performed on Varian™ CP-3800 gas chromatograph coupled with a Varian™ Saturn® 2000 mass spectrometer. Standards were dissolved in EtOAc and biotransformations were extracted with EtOAc. 10 µL of sample was injected onto an Agilent J&W HP-5 (30 mm x 0.32 mm i.d. x 0.25 µm film thickness) column at 200 °C. The oven temperature was set at 40 °C for 1 min and raised at 5 °C/min to 100 °C, then at 2 °C/min to 150 °C, finally at 10 °C /min to 300 °C. The carrier gas flow rate was 1.0 mL/min. The mass spectrometer was operated in the full scan mode. The following gas chromatograph parameters were used: detector temperature was set at 270 °C.

**GC-MS method B**: GC-MS analysis were performed on an Agilent™ 7890B gas chromatograph coupled with an Agilent™ 5977A mass spectrometer. Standards were dissolved in EtOAc and biotransformations were extracted with EtOAc. 10 µL of sample was injected onto an Agilent J&W HP-1 (30 mm x 0.32 mm i.d. x 0.25 µm film thickness) column at 200 °C. The oven temperature was set at 50 °C for 1 min and raised at 5 °C/min to 120 °C and held for 1 min, then at 10 °C/min to 320 °C. The carrier gas flow rate was 2.0 mL/min. The mass spectrometer was operated in the full scan mode. The following gas chromatograph parameters were used: detector temperature was set at 270 °C, Split ratio 100: 1, pressure 4.58 psi and velocity 52 cm/sec.

**Table 2.** Retention time table for GC-MS analysis

| Compound | GC Method | Retention time (min) |
|---|---|---|
| **2** | GC-MS method A | 4.3 |
| **7** | GC-MS method B | 9.4 |
| **a** | GC-MS method A | 5.6 |
| | GC-MS method B | 4.2 |
| **2a** | GC-MS method A | 32.7 |
| **7a** | GC-MS method B | 24.6 |
| **aminotetralin** | GC-MS method A | 22.7 |
| **Tetralone** | GC-MS method A | 24.3 |

### 3.2 GC-MS traces

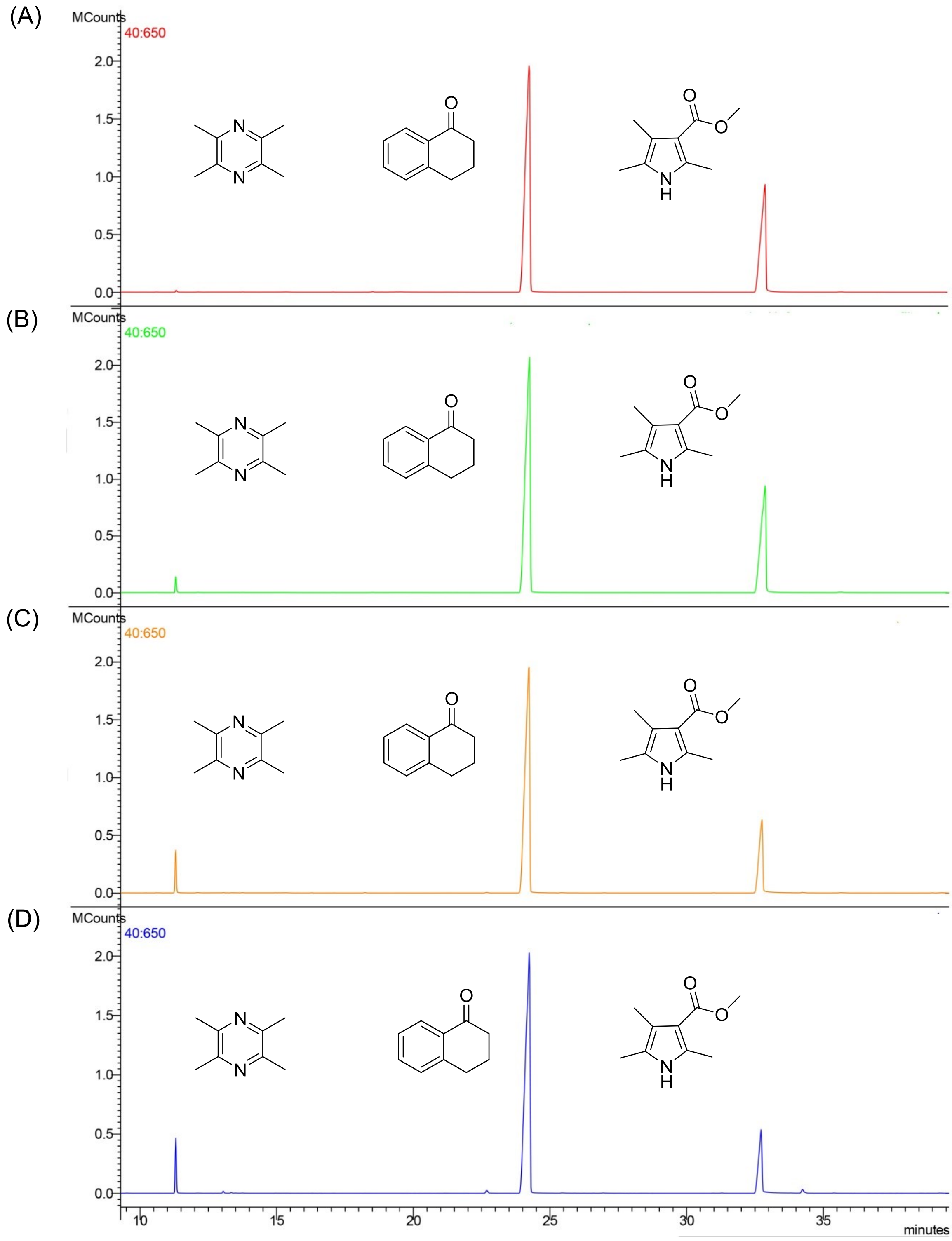


**Figure S8**: GC-MS traces for the conversion of α-diketone **2** to pyrrole **2b** and pyrazine **5** in the presence of: (A) 10 equiv of β-keto ester **b,** pH 5; (B) 10 equiv of β-keto ester **b**, pH 6; (C) 10 equiv of β-keto ester **b**, pH 7.4;(D) 10 equiv of β-keto ester **b**, pH 9.

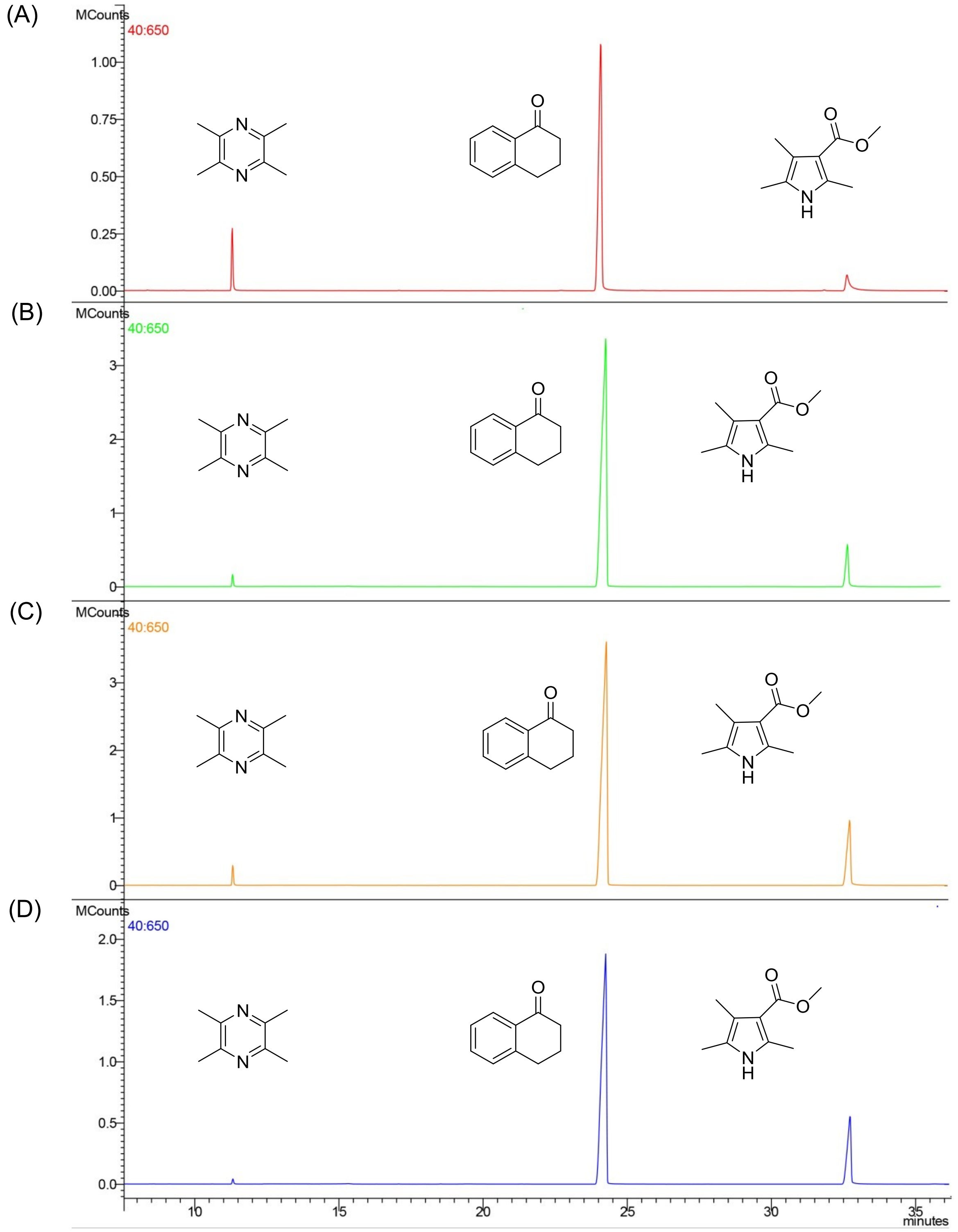


**Figure S9**: GC-MS traces for the conversion of α-diketone **2** to pyrrole **2b** and pyrazine **5** in the presence of: (A) 3 equiv of β-keto ester **b**, pH 7.4; (B) 1 equiv of β-keto ester **b**, pH 5; (C) 2 equiv of β-keto ester **b**, pH 5; (D) 3 equiv of β-keto ester **b**, pH 5.

(A)

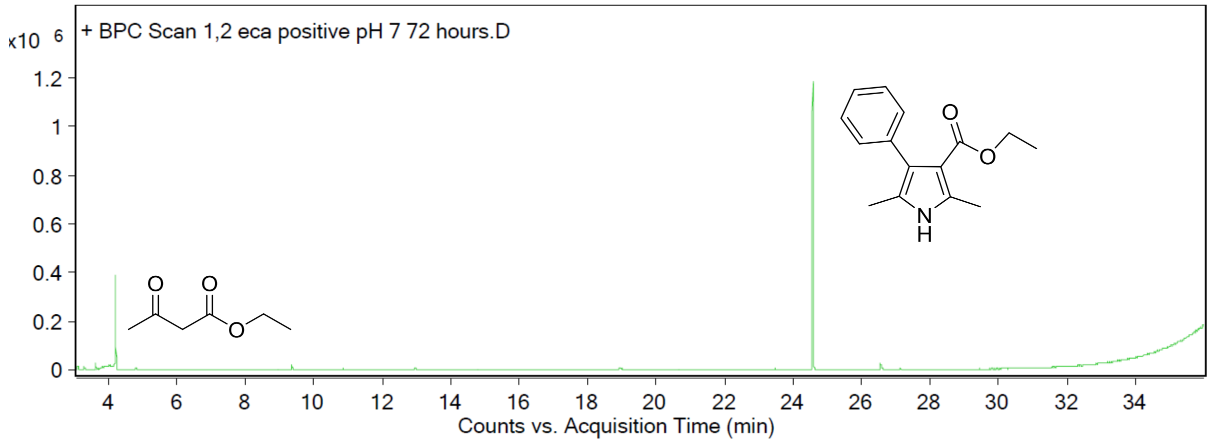


(B)

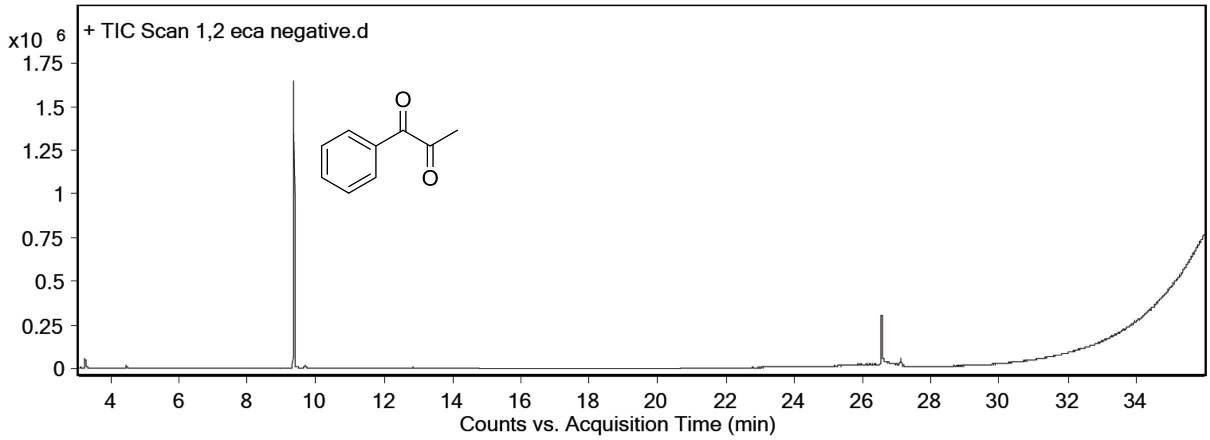


**Figure S10**: GC-MS traces for the conversion of β-amino ester **13** and α-diketone **7** to pyrrole **7a**: (A) in the presentce of ATA-117 and (B) in the absence of enzyme. No product was detected in the no enzyme control.

# 4. NMR analysis

## 4.1 NMR spectra of chemical standards and isolated chemicals

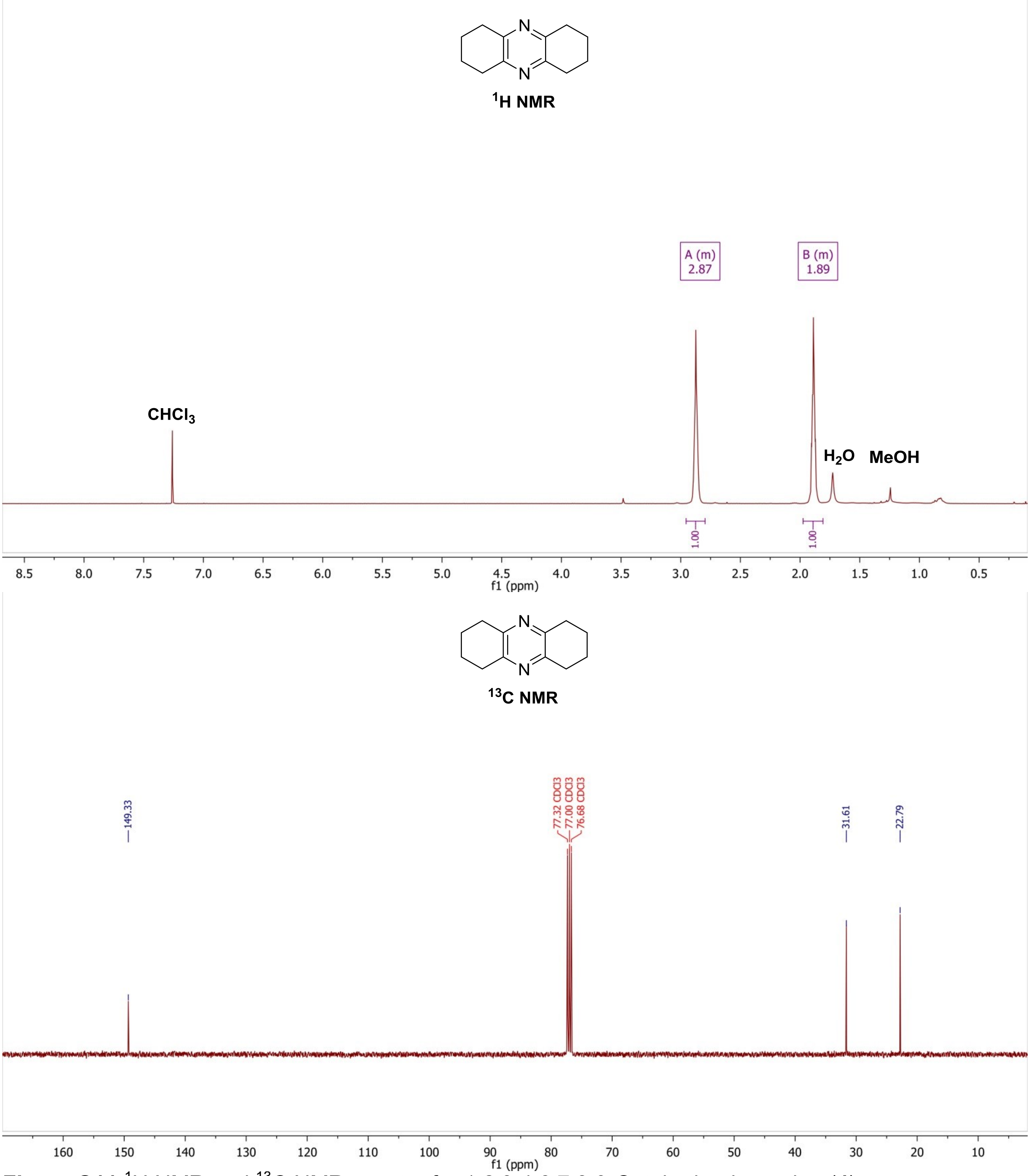


**Figure S11**:$^{1}$H NMR and $^{13}$C NMR spectra for 1,2,3,4,6,7,8,9-Octahydrophenazine (**4**).

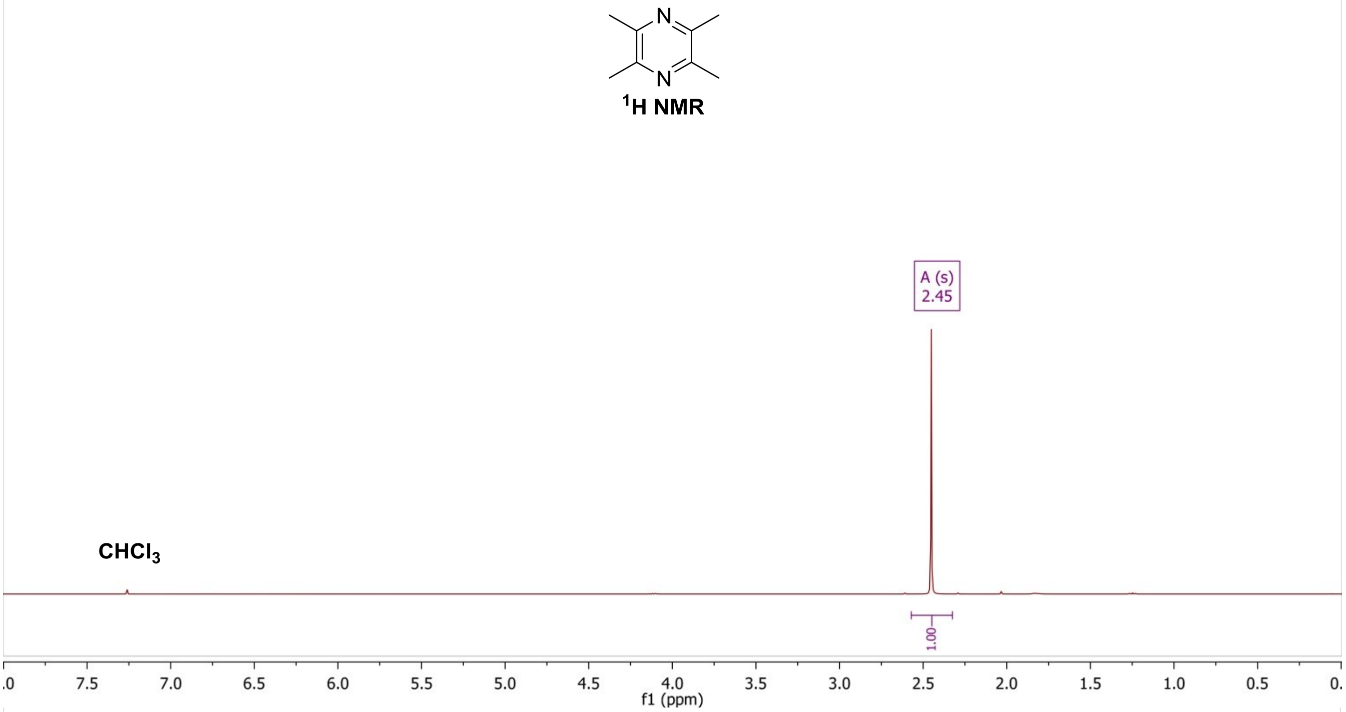


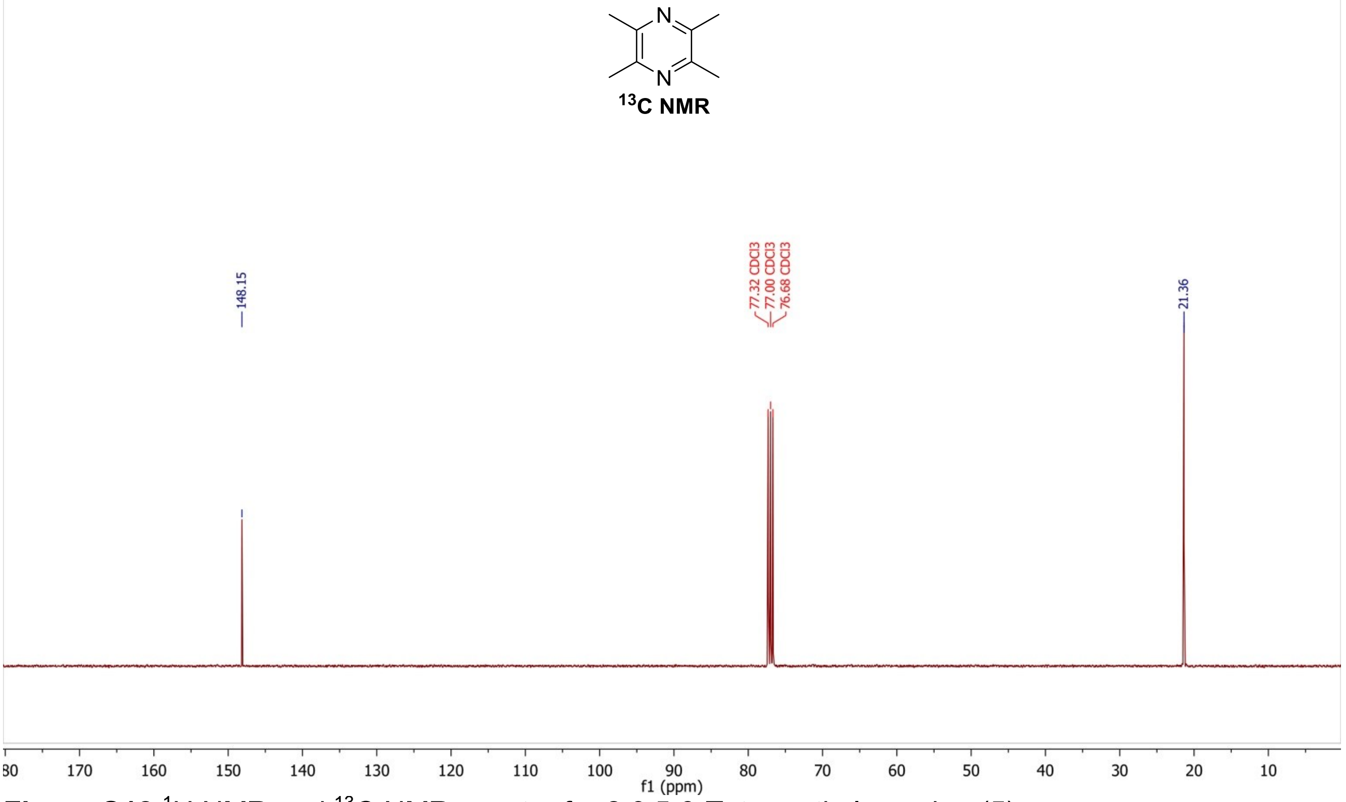


**Figure S12**:$^1$H NMR and $^{13}$C NMR spectra for 2,3,5,6-Tetramethylpyrazine (**5**).

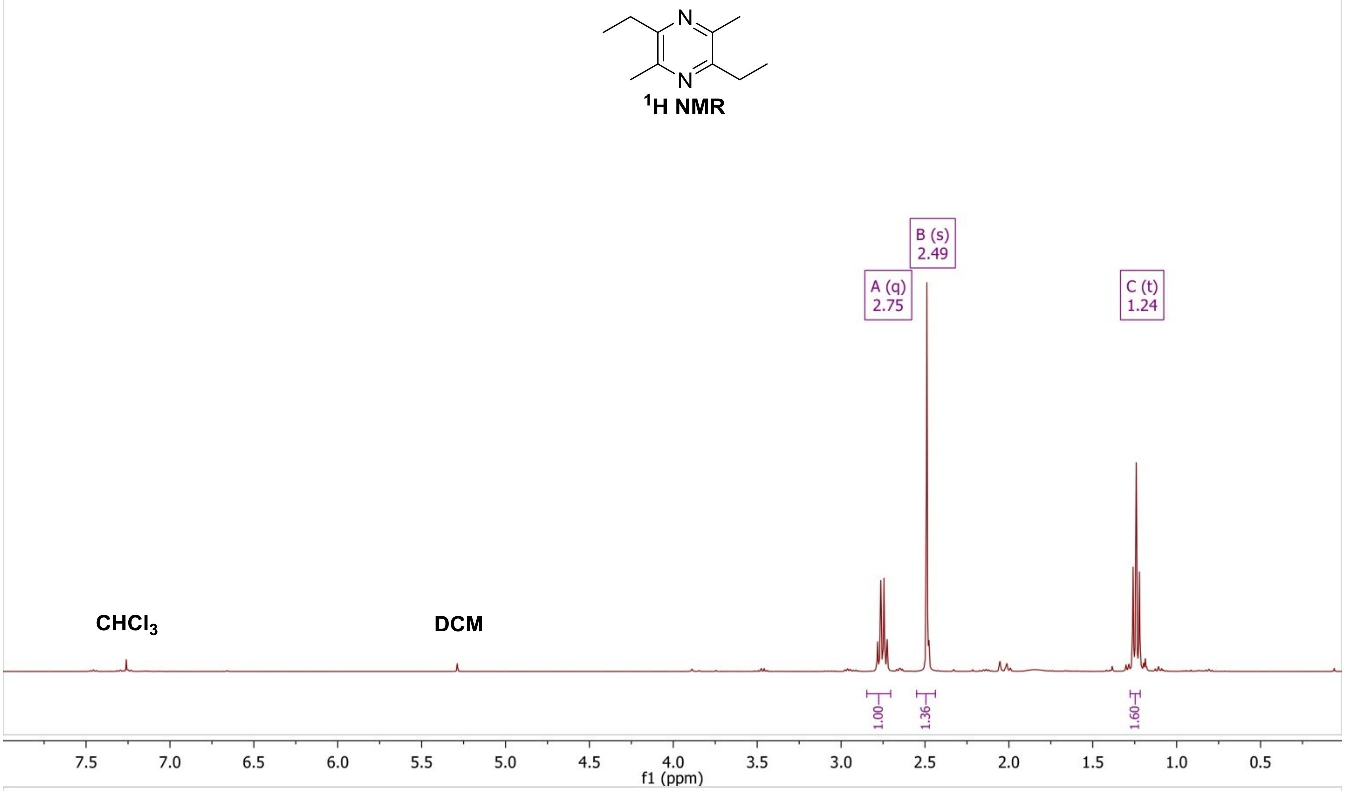


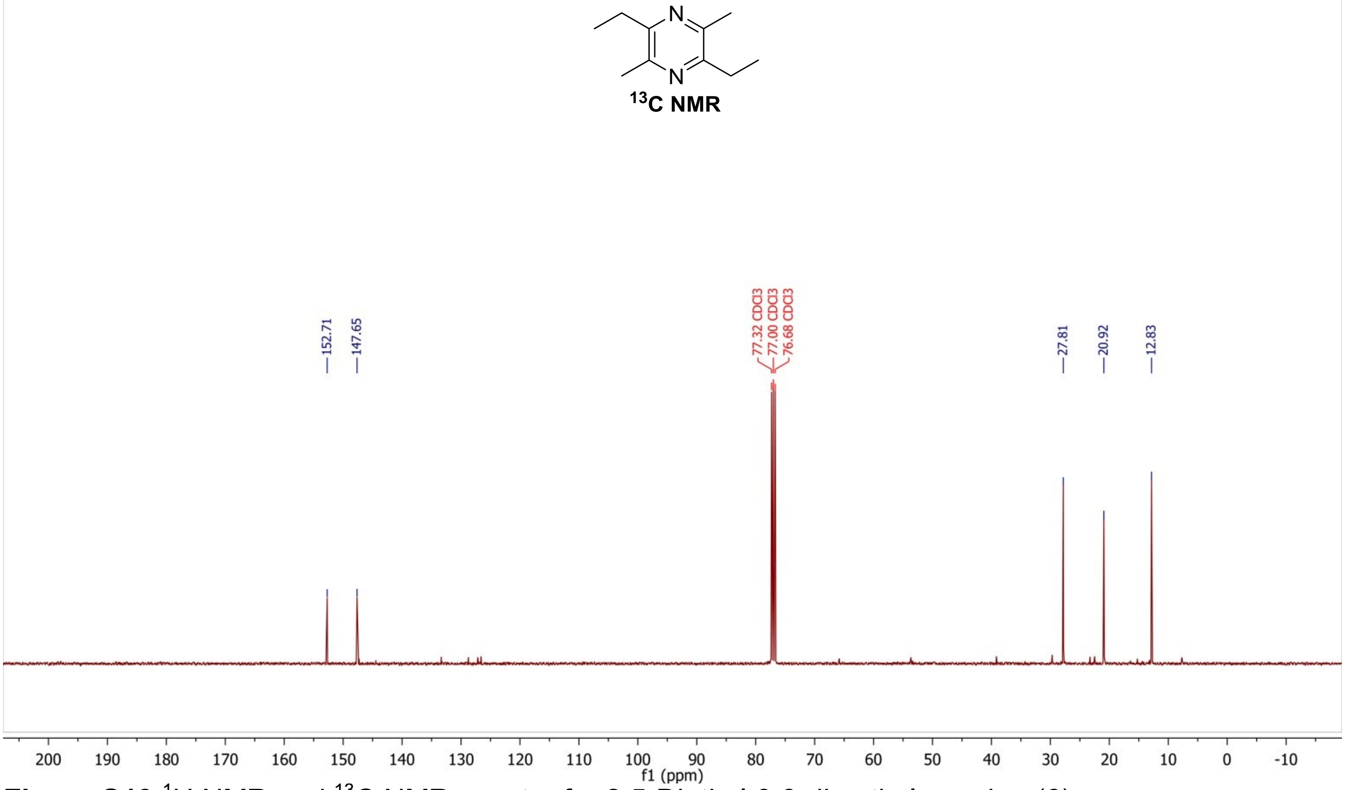


**Figure S13**:$^1$H NMR and $^{13}$C NMR spectra for 2,5-Diethyl-3,6-dimethylpyrazine (**6**).

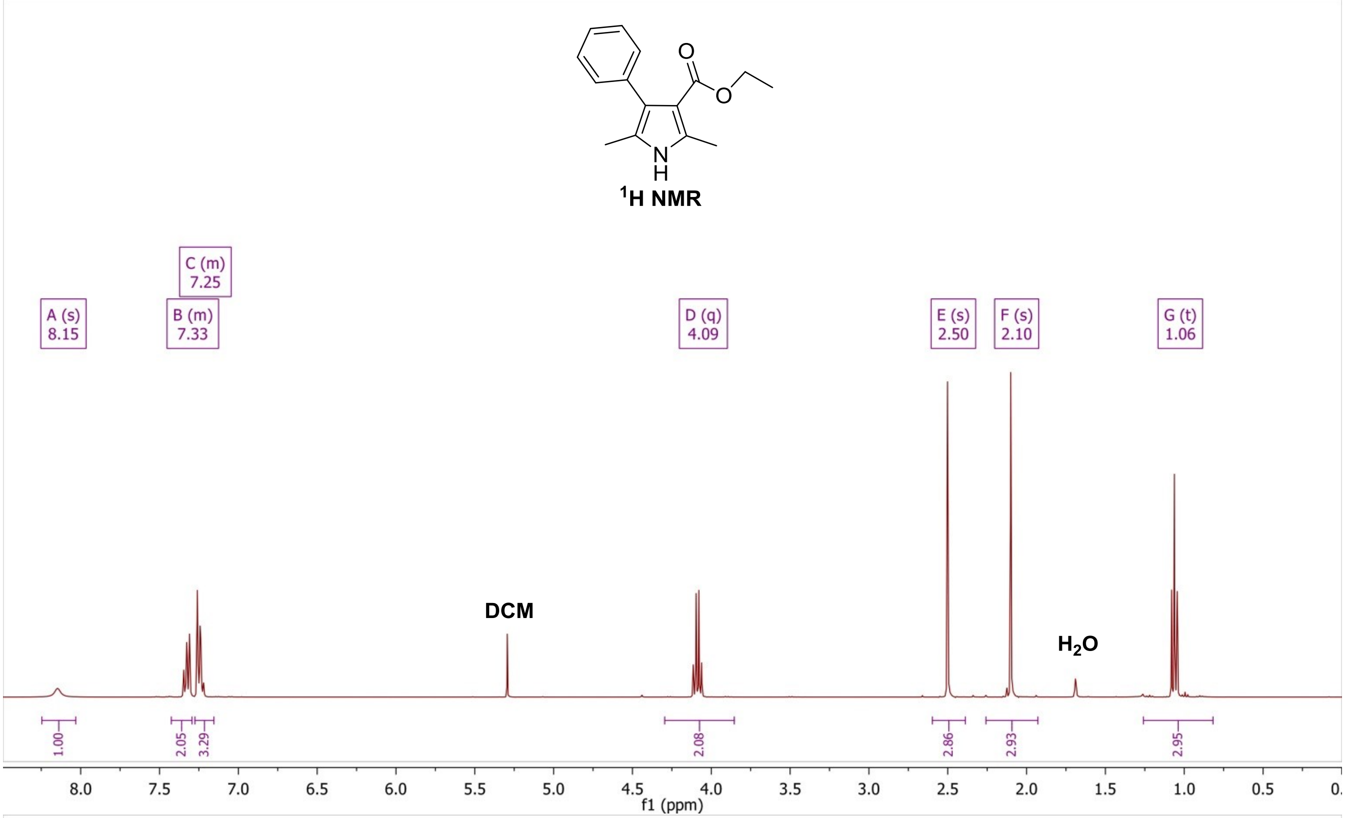


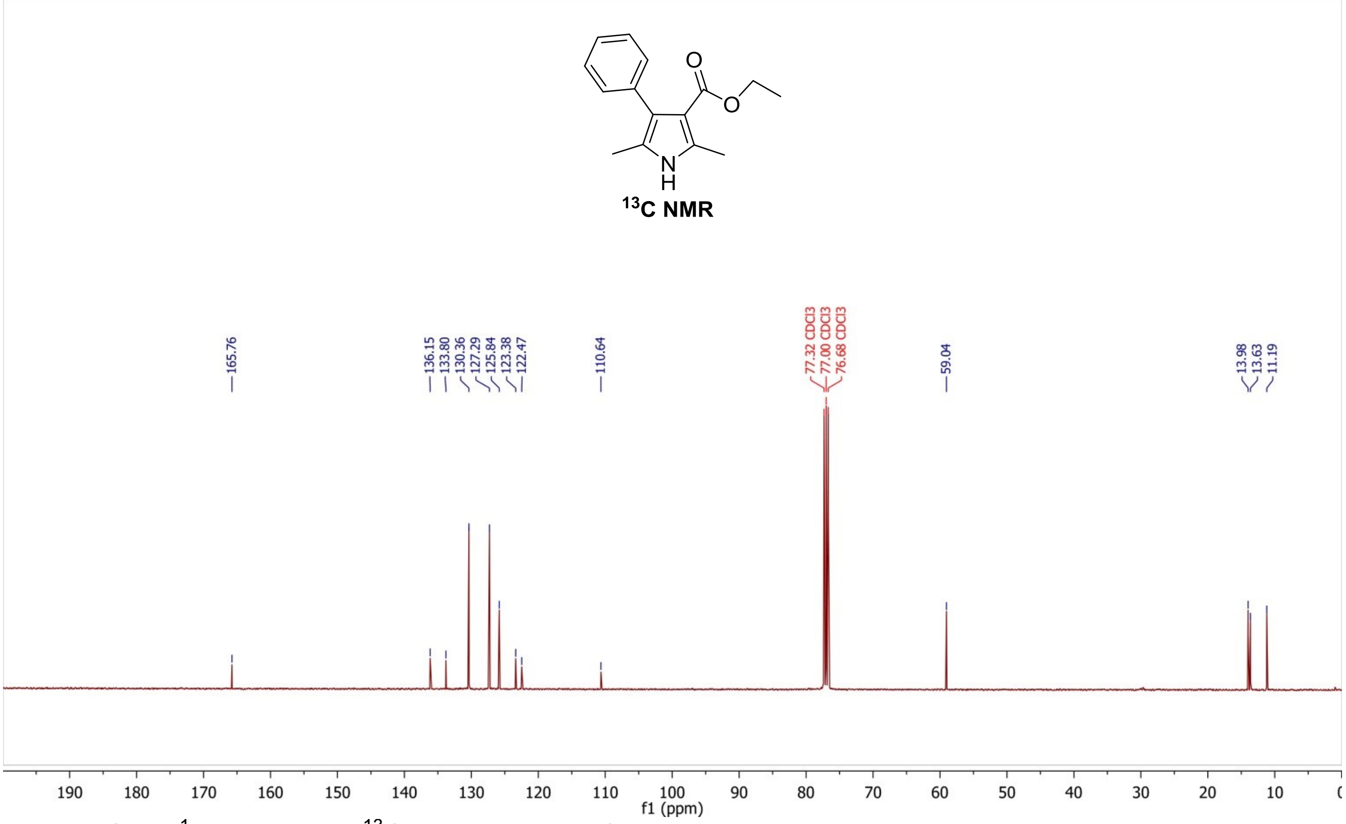


**Figure S14**: $^1$H NMR and $^{13}$C NMR spectra for Ethyl 2,5-dimethyl-4-phenyl-1*H*-pyrrole-3-carboxylate (**7a**).

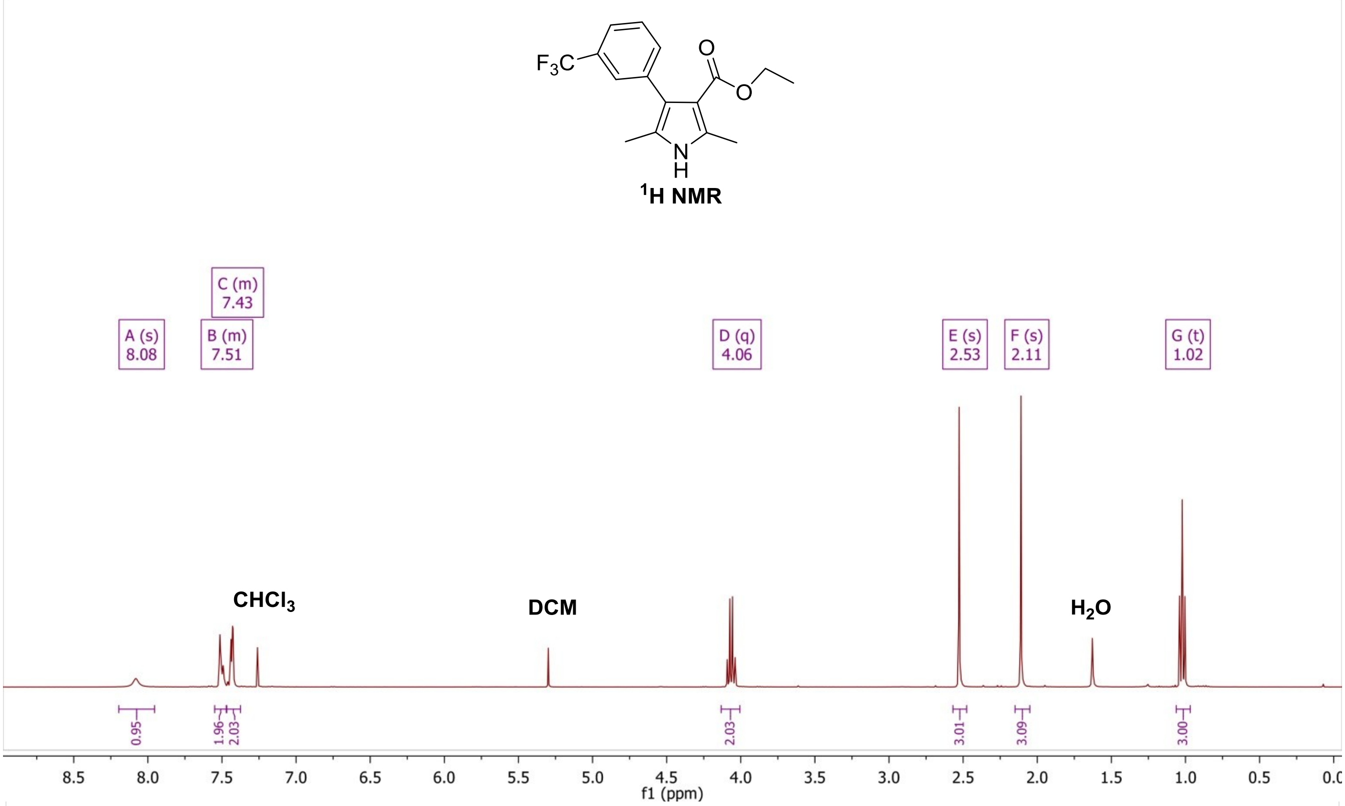


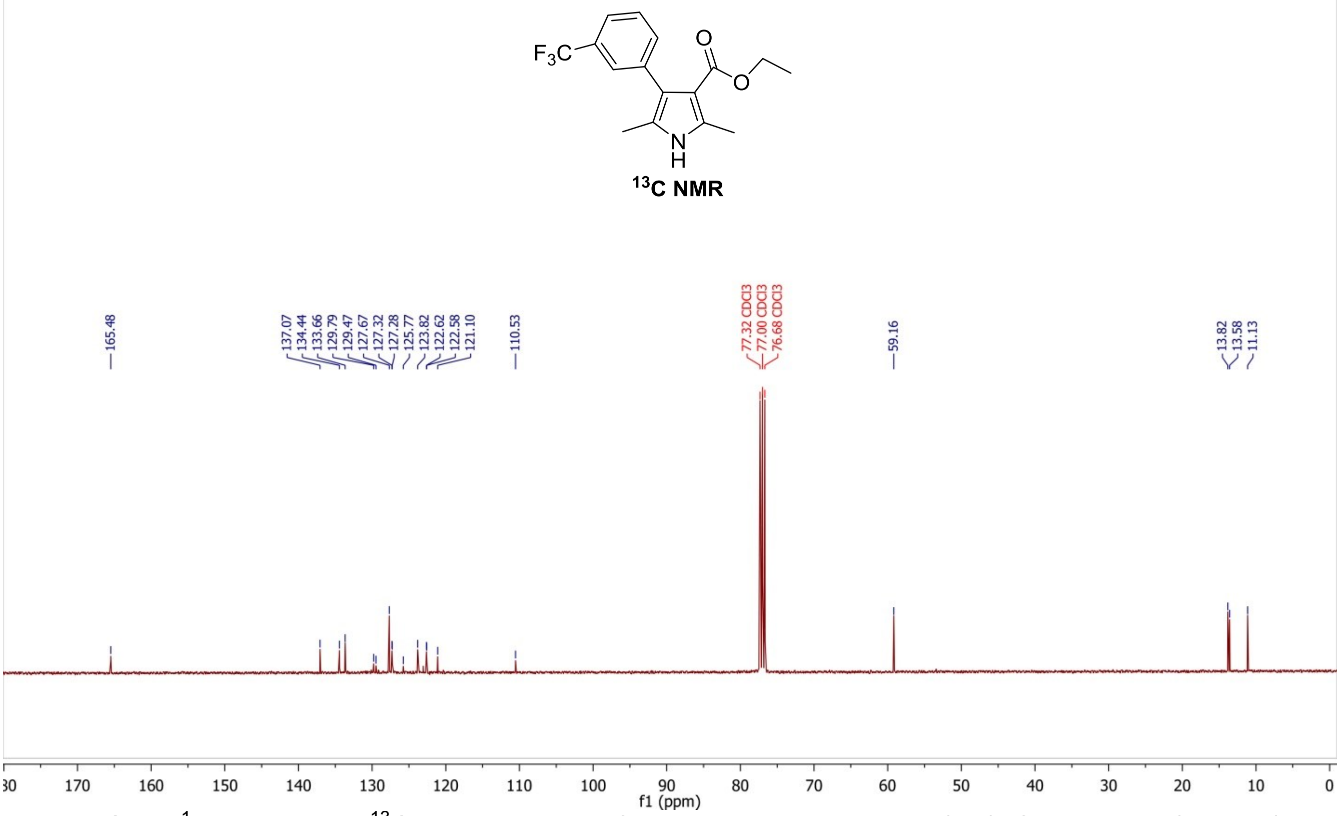


**Figure S15**: $^1$H NMR and $^{13}$C NMR spectra for Ethyl 2,5-dimethyl-4-(3'-(trifluoromethyl)phenyl)-1*H*-pyrrole-3-carboxylate (**8a**).

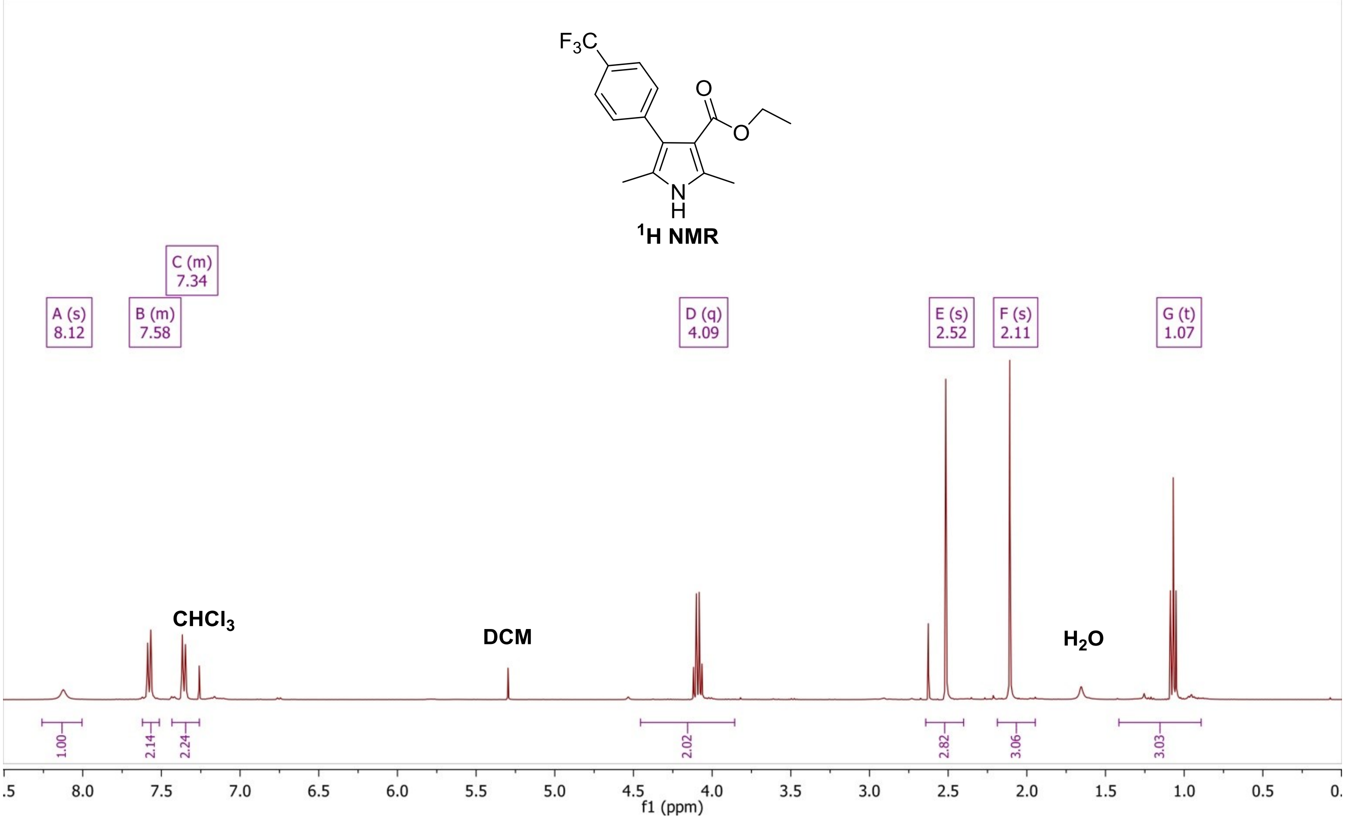


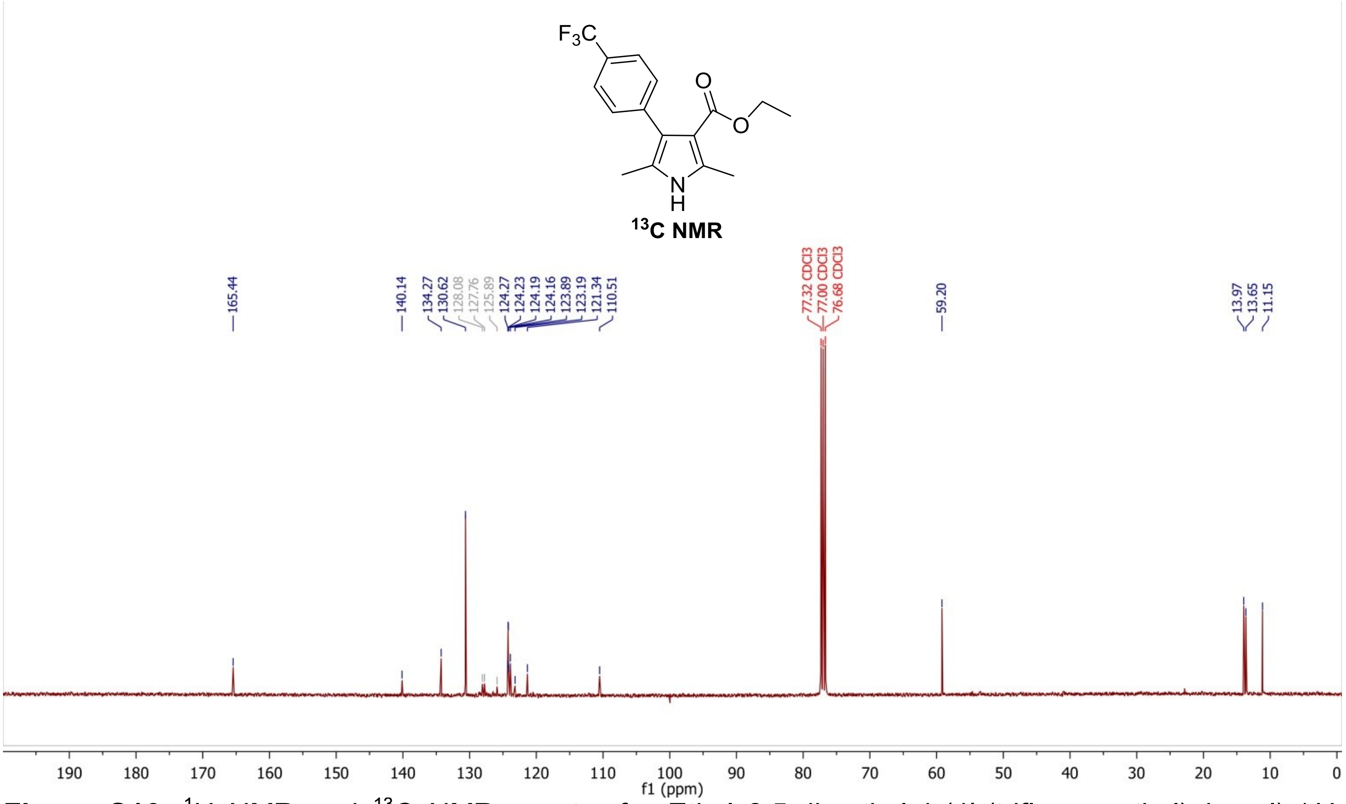


**Figure S16**: $^{1}H$ NMR and $^{13}C$ NMR spectra for Ethyl 2,5-dimethyl-4-(4'-(trifluoromethyl)phenyl)-1*H*-pyrrole-3-carboxylate (**9a**).

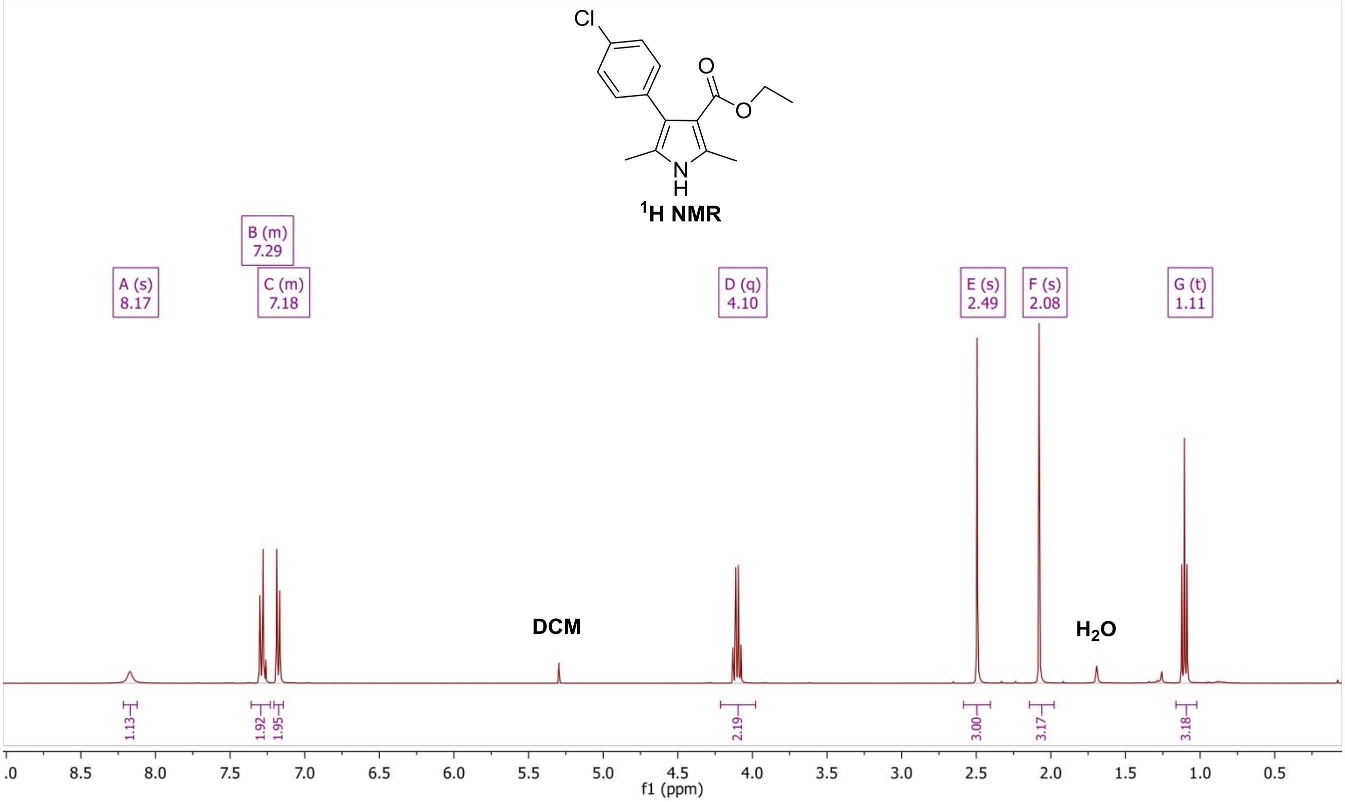


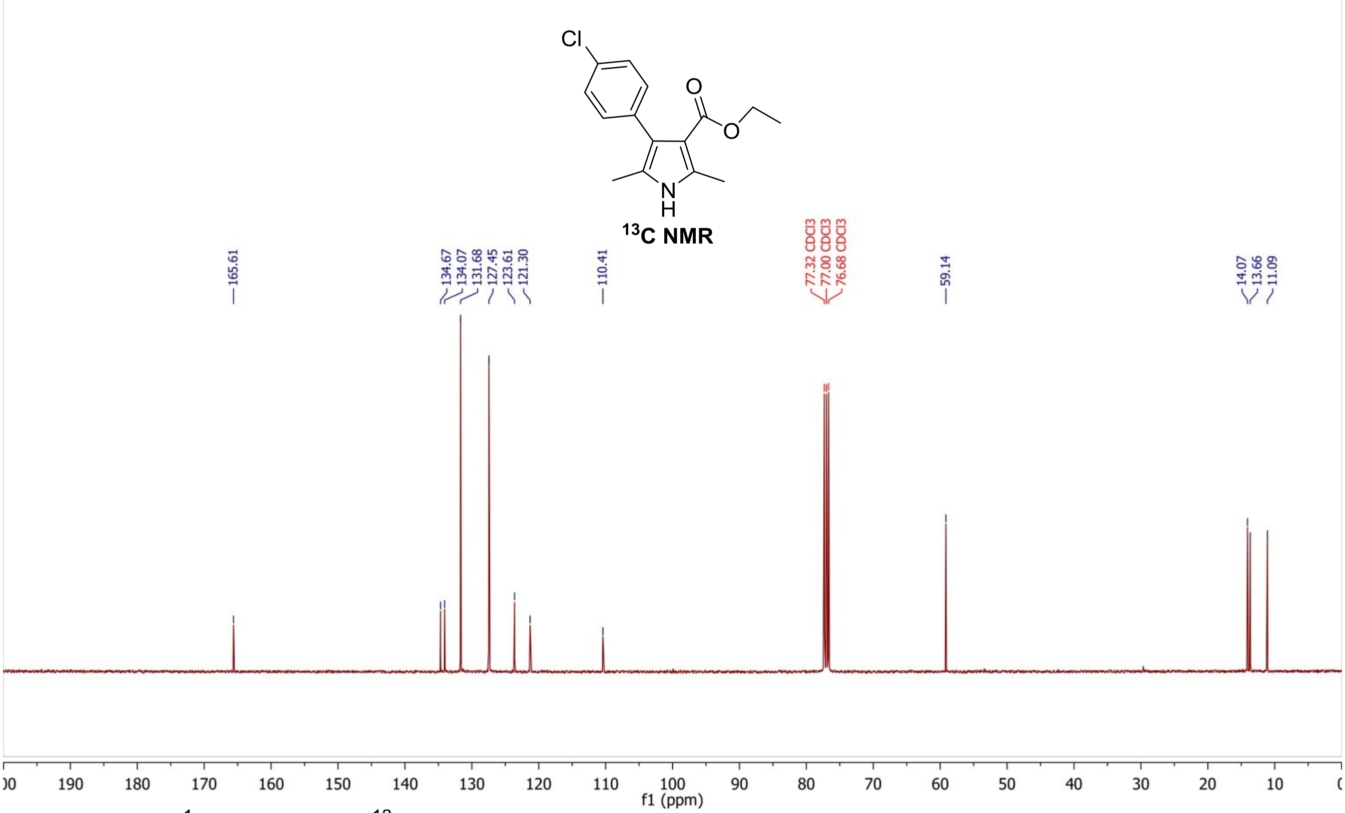


**Figure S17**: $^1$H NMR and $^{13}$C NMR spectra for Ethyl 2,5-dimethyl-4-(4'-chlorophenyl)-1*H*-pyrrole-3-carboxylate (**10a**).

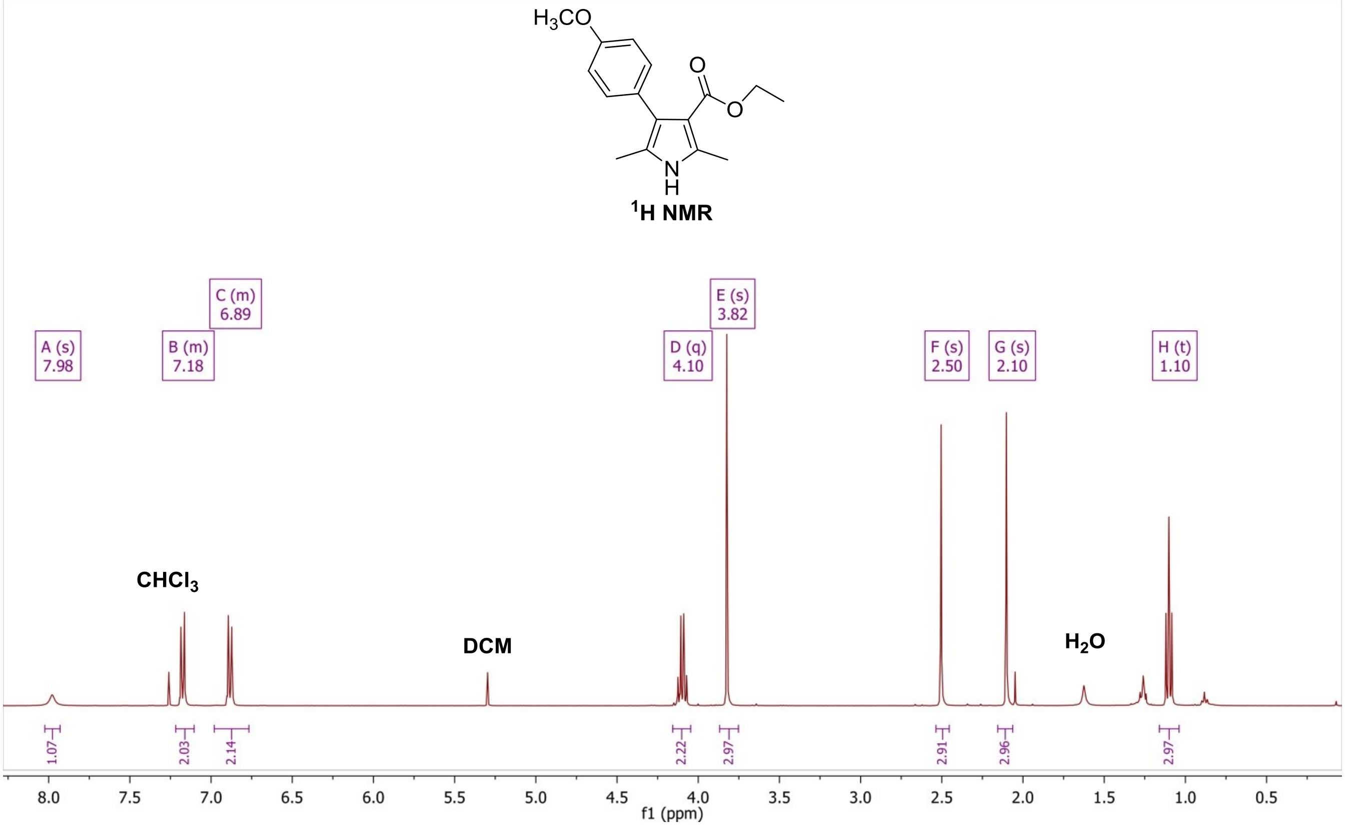


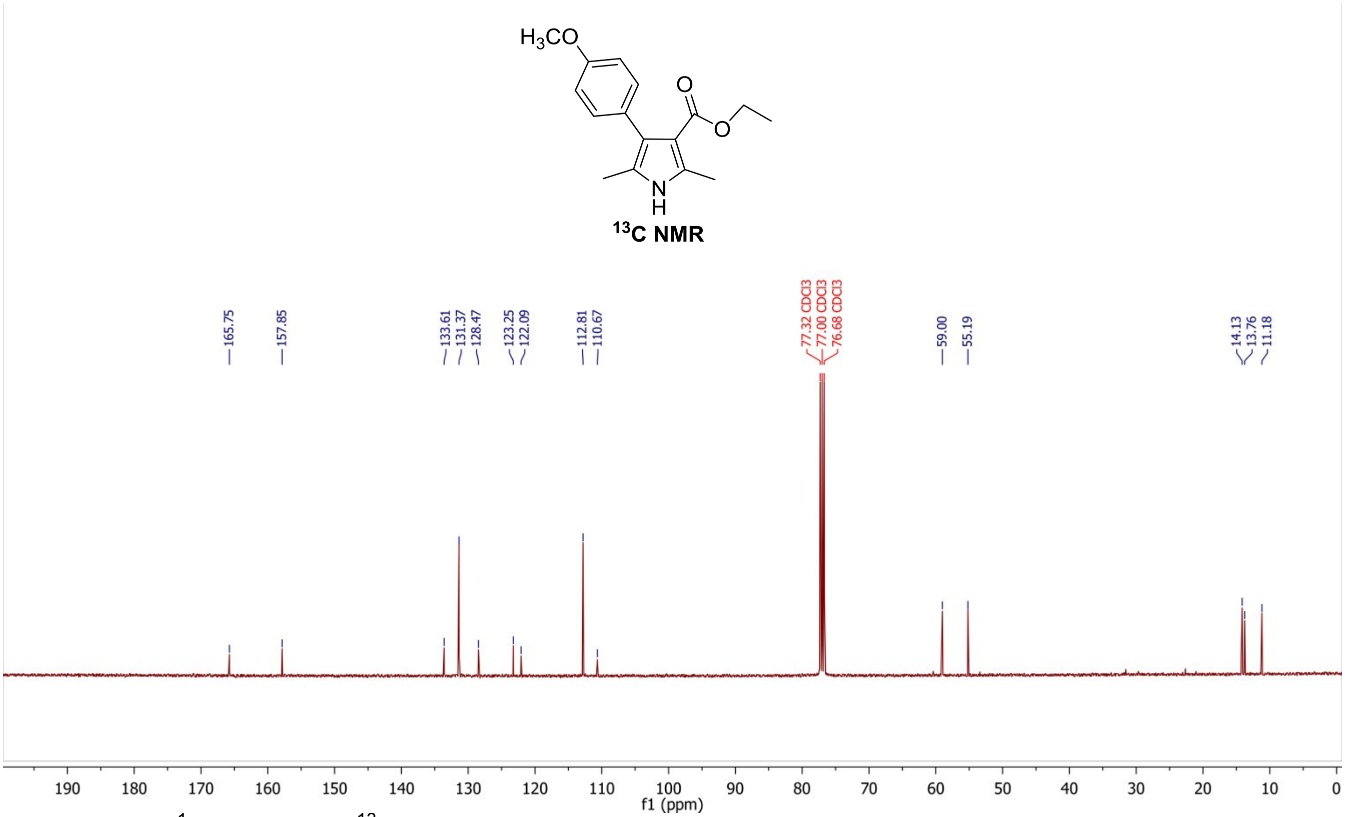


**Figure S18**: $^{1}H$ NMR and $^{13}C$ NMR spectra for Ethyl 2,5-dimethyl-4-(4'-methoxyphenyl)-1*H*-pyrrole-3-carboxylate (**11a**).

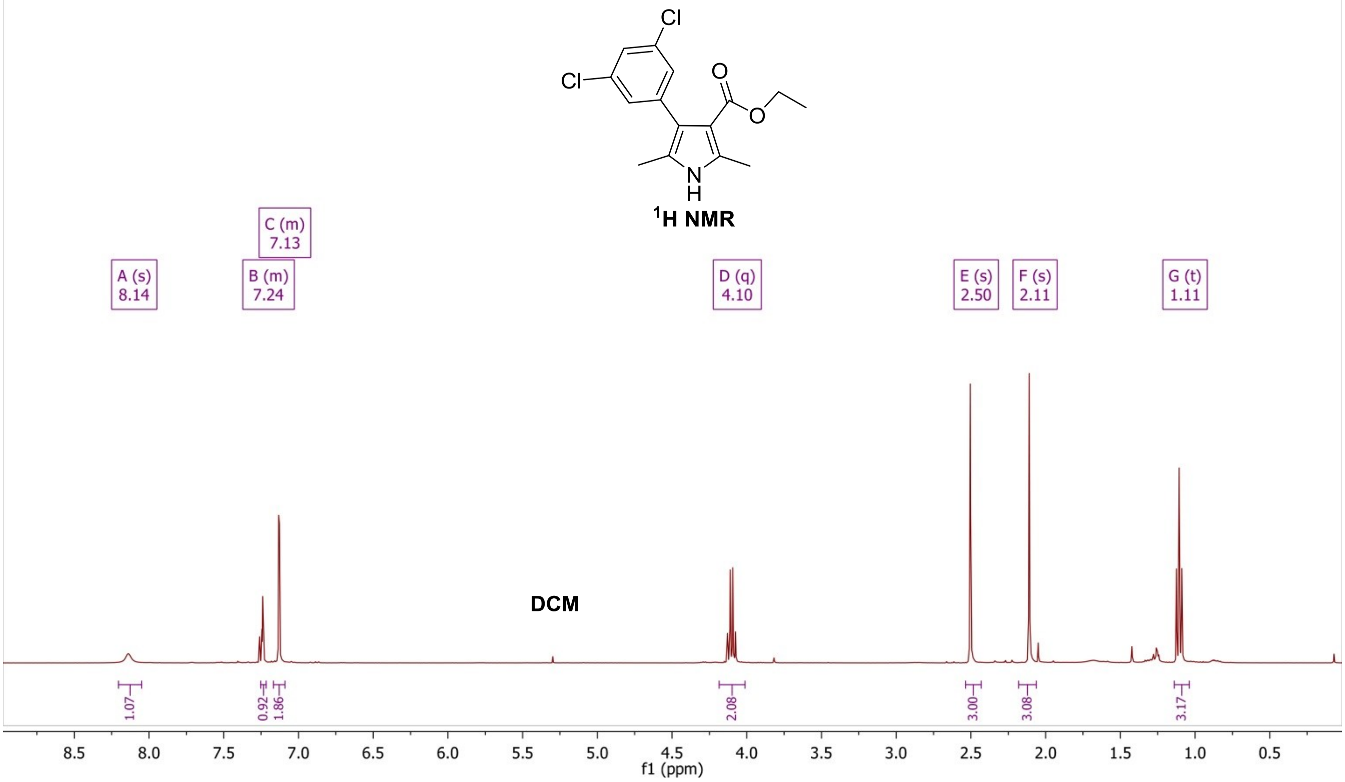


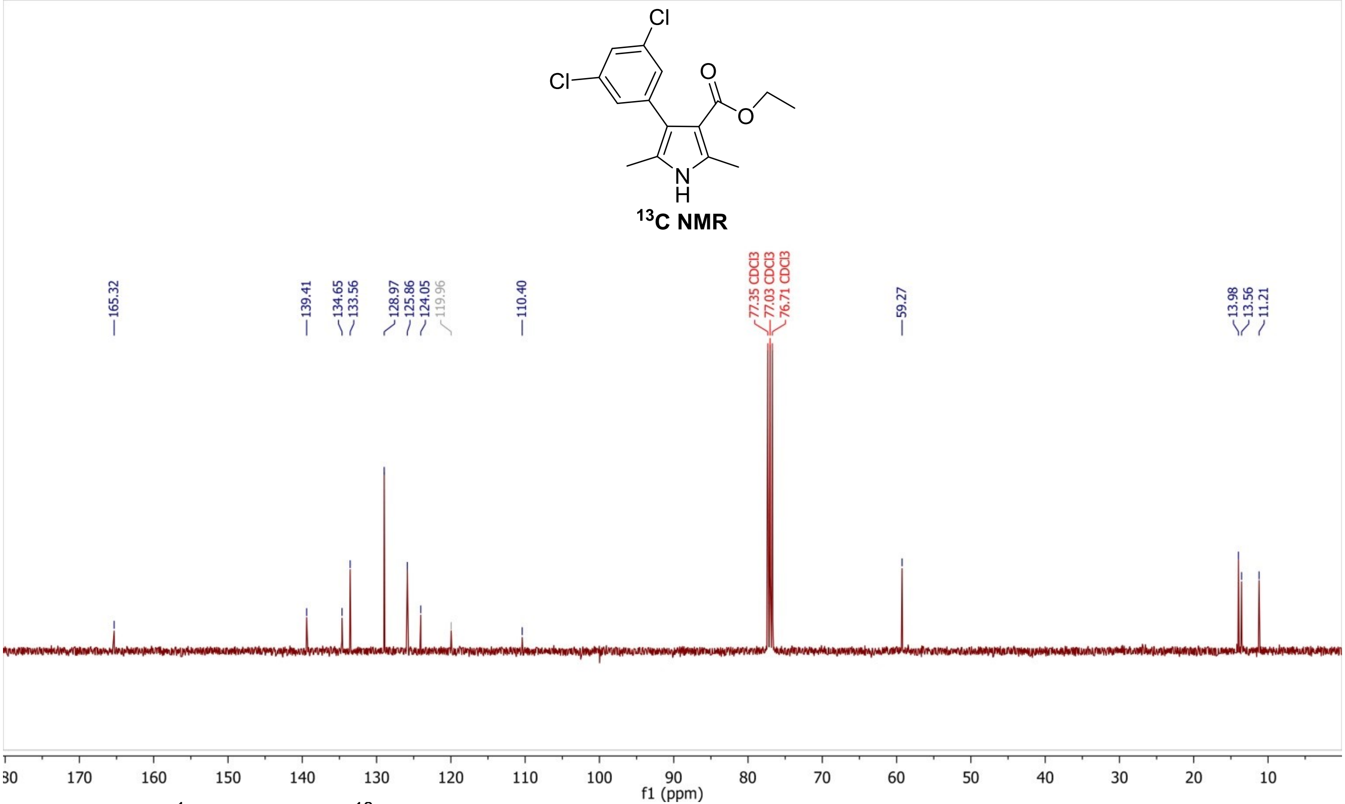


**Figure S19**: $^{1}$H NMR and $^{13}$C NMR spectra for Ethyl 2,5-dimethyl-4-( 3',5'-dichlorophenyl)-1*H*-pyrrole-3-carboxylate (**12a**).

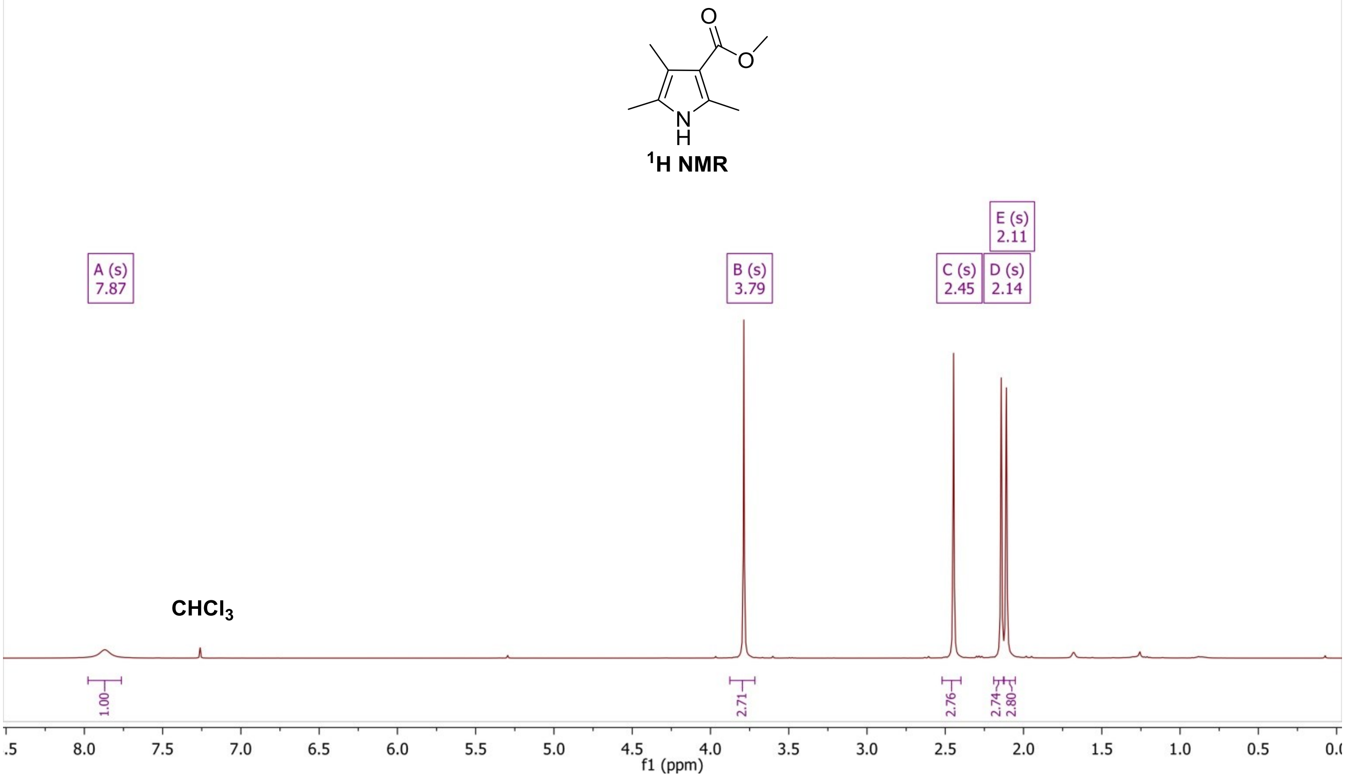


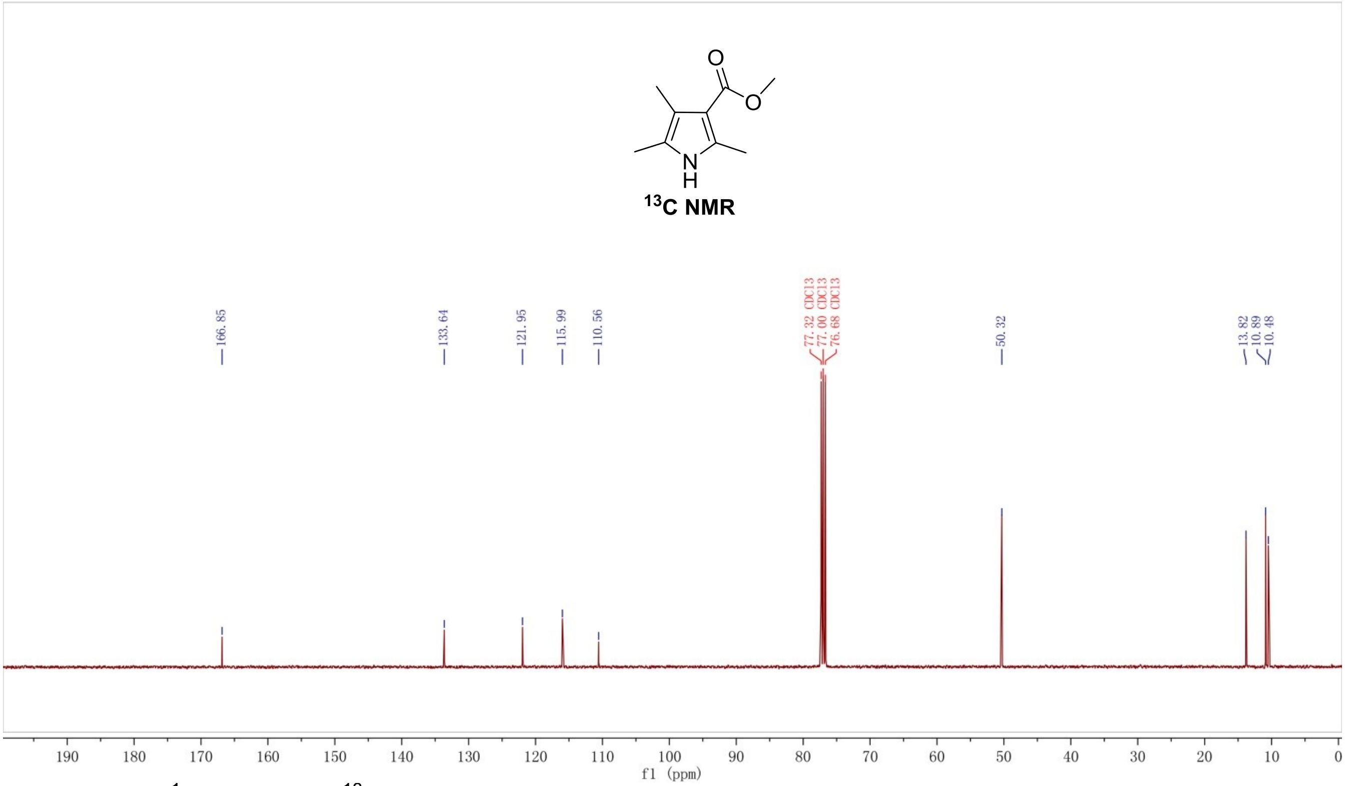


**Figure S20**: $^{1}$H NMR and $^{13}$C NMR spectra for Methyl 2,4,5-trimethyl-1*H*-pyrrole-3-carboxylate (**2b**).

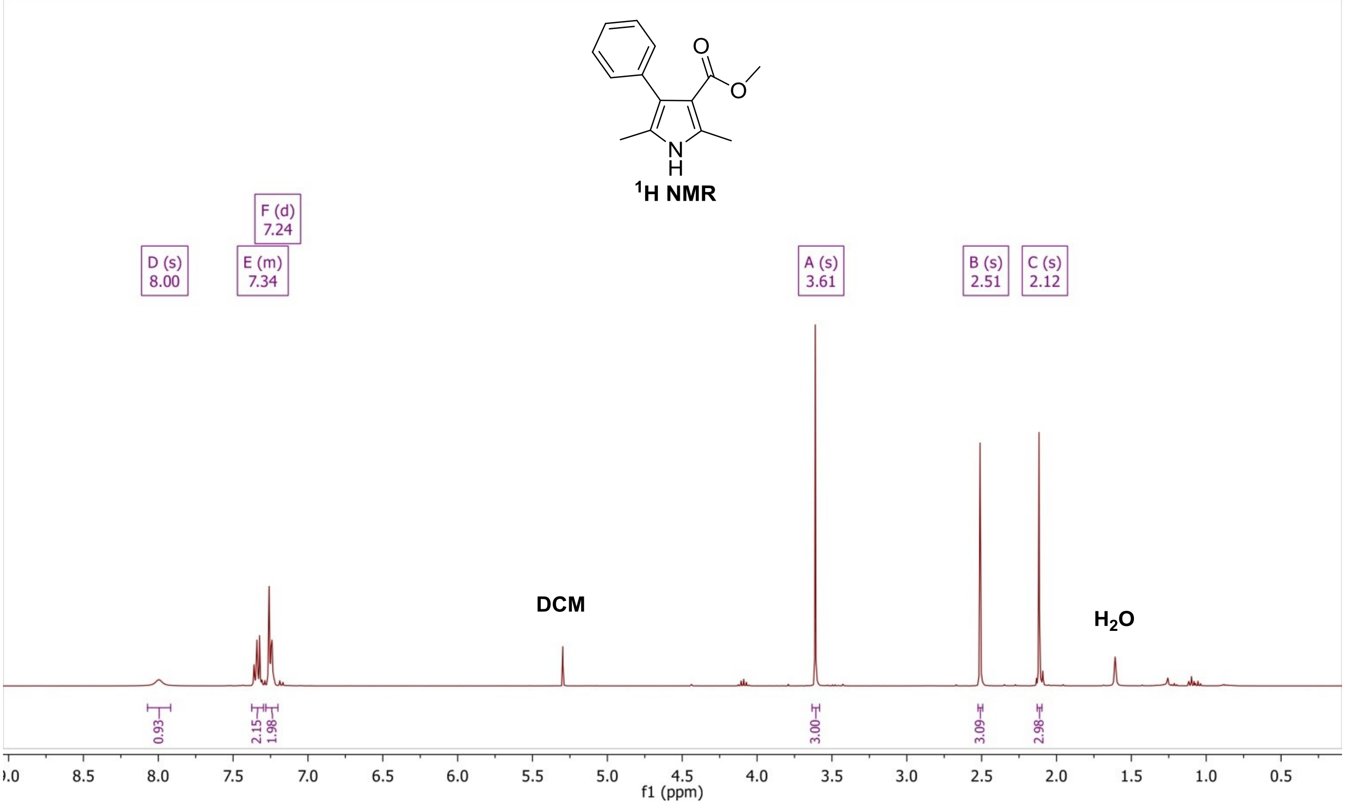


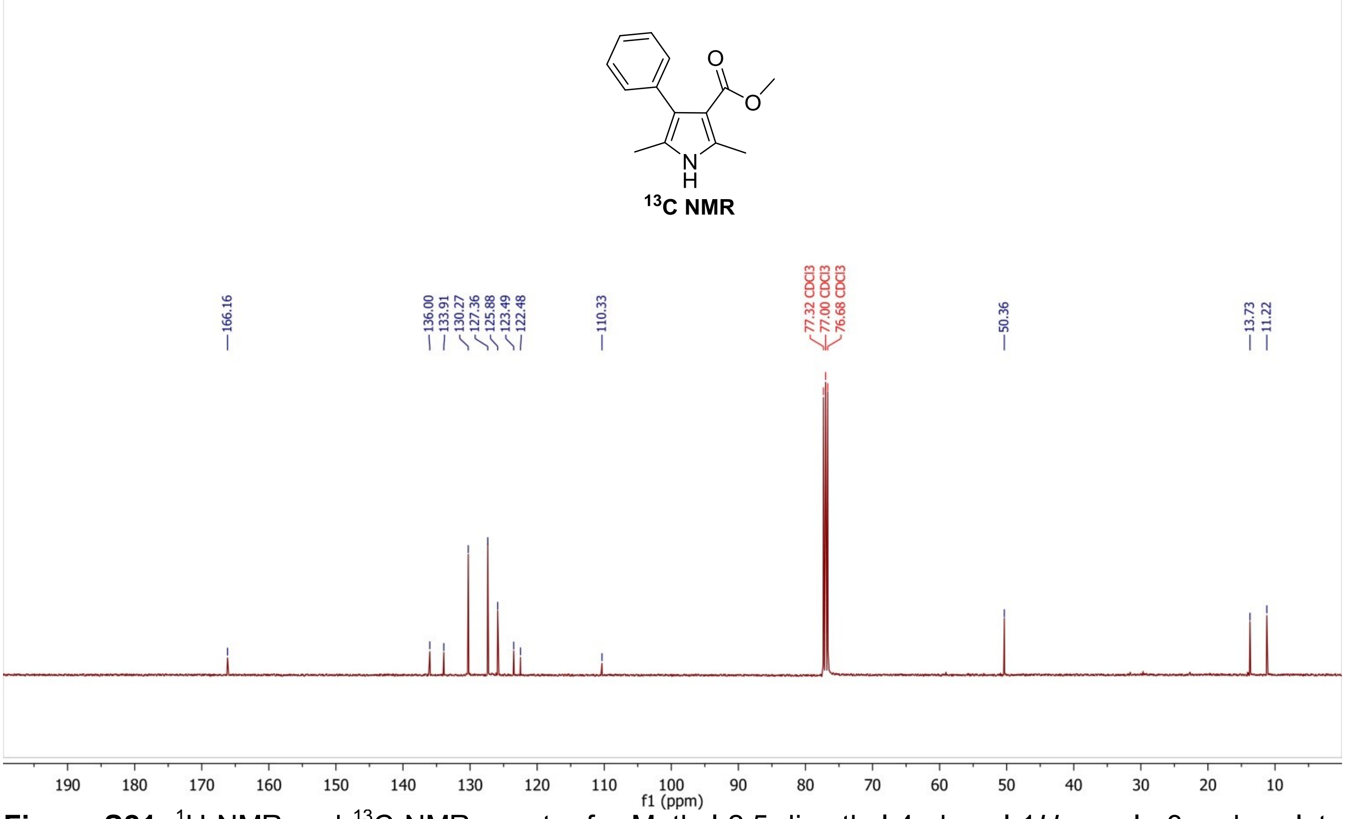


**Figure S21**: $^{1}H$ NMR and $^{13}C$ NMR spectra for Methyl 2,5-dimethyl-4-phenyl-1*H*-pyrrole-3-carboxylate (**7b**).

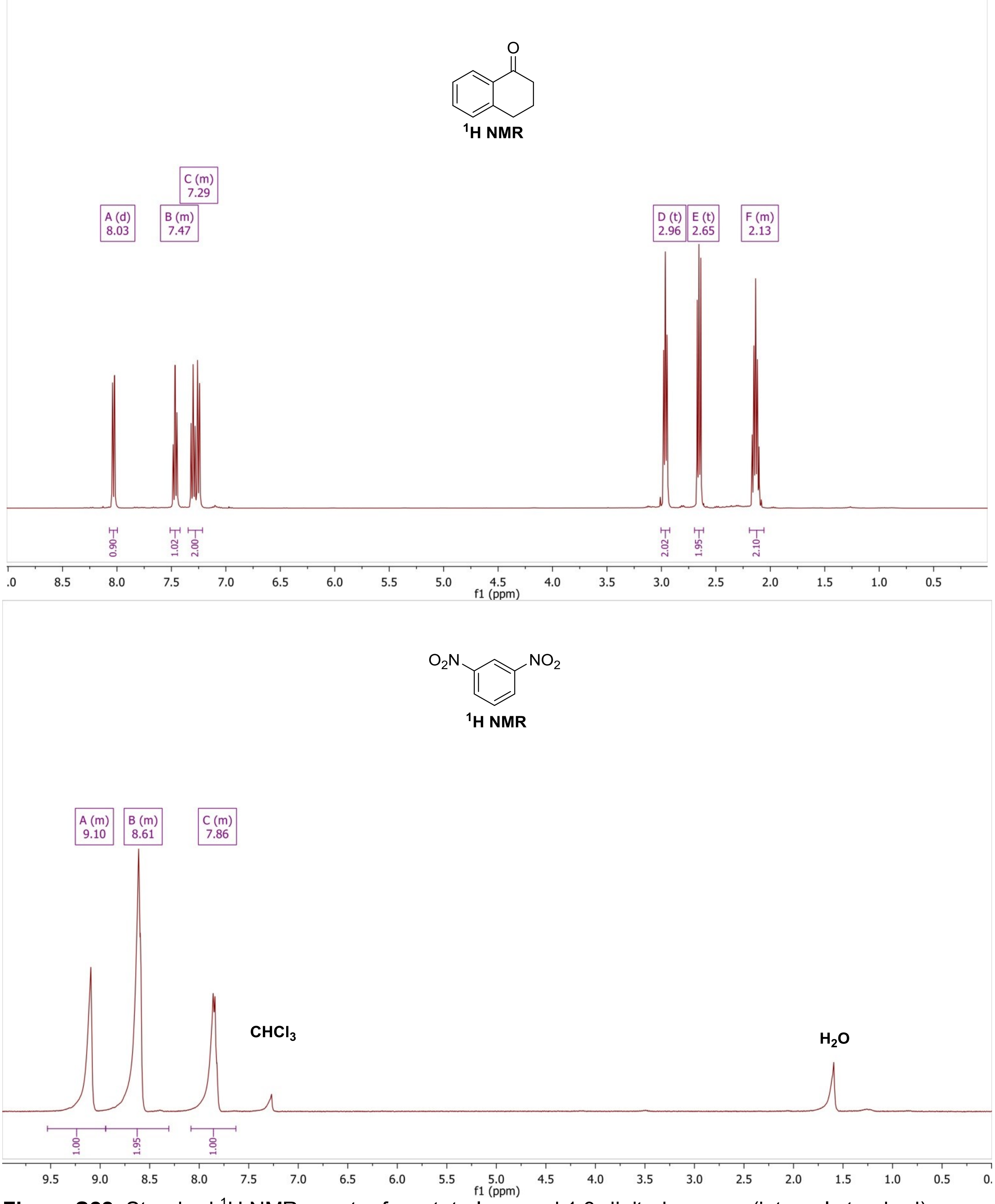


**Figure S22**: Standard $^{1}$H NMR spectra for α-tetralone and 1,3-dinitrobenzene (internal standard).

## 5. Mass spectra

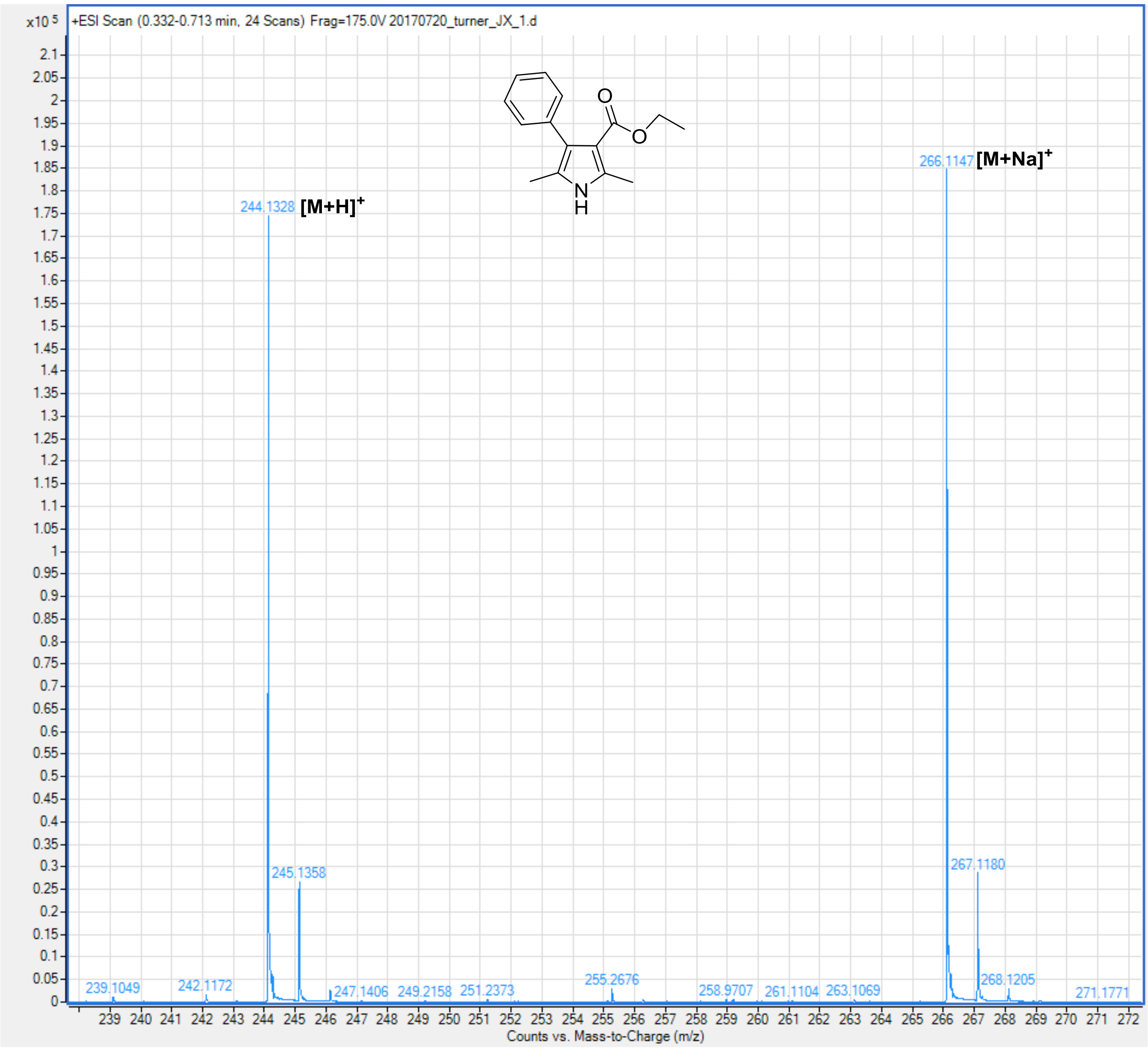


**Figure S23**: HRMS spectrum for Ethyl 2,5-dimethyl-4-phenyl-1*H*-pyrrole-3-carboxylate (**7a**)

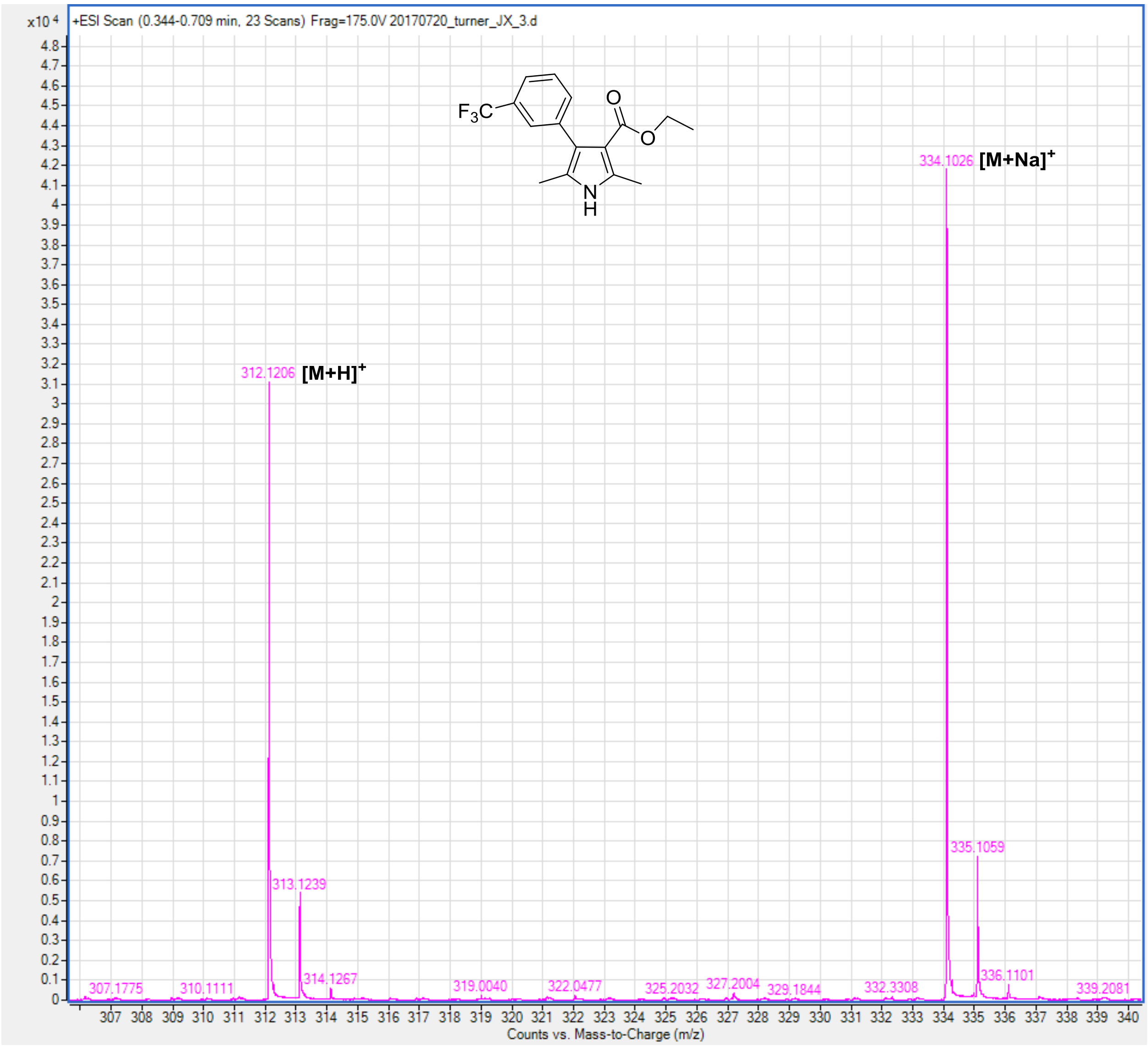


**Figure S24**: HRMS spectrum for Ethyl 2,5-dimethyl-4-(3'-(trifluoromethyl)phenyl)-1*H*-pyrrole-3-carboxylate (**8a**)

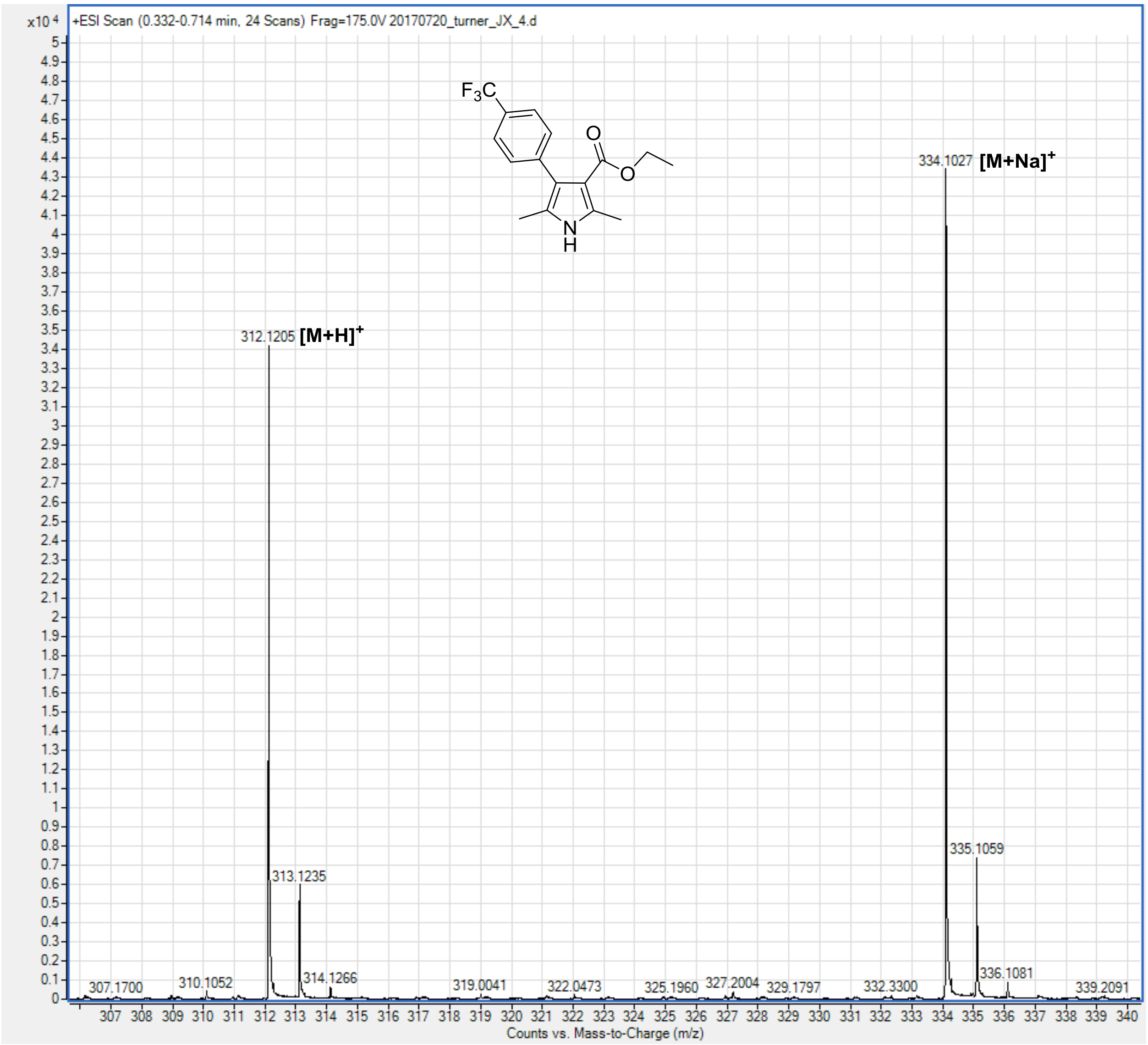


**Figure S25**: HRMS spectrum for Ethyl 2,5-dimethyl-4-(4'-(trifluoromethyl)phenyl)-1*H*-pyrrole-3-carboxylate (**9a**)

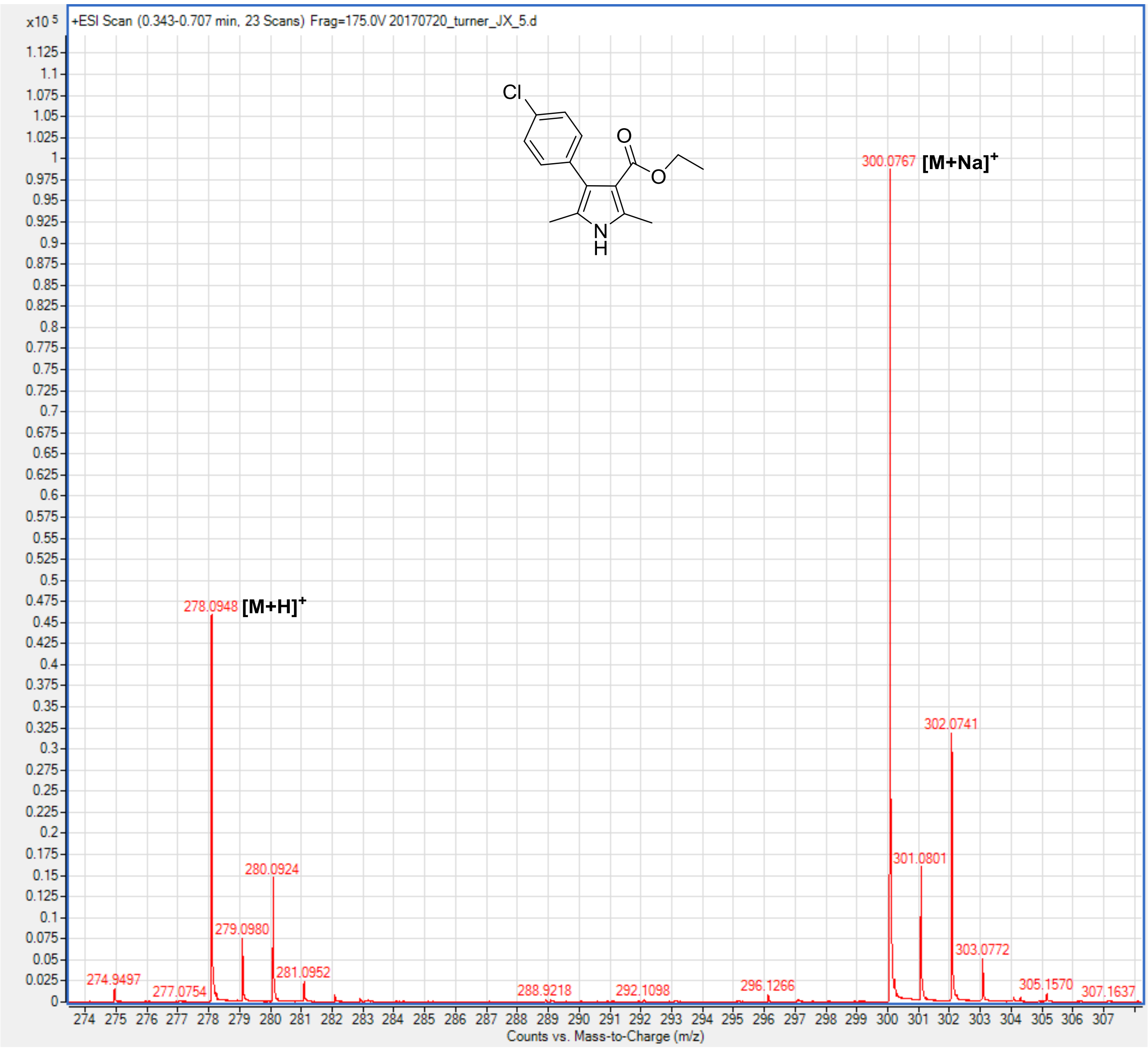


**Figure S26**: HRMS spectrum for Ethyl 2,5-dimethyl-4-(4'-chlorophenyl)-1*H*-pyrrole-3-carboxylate (**10a**)

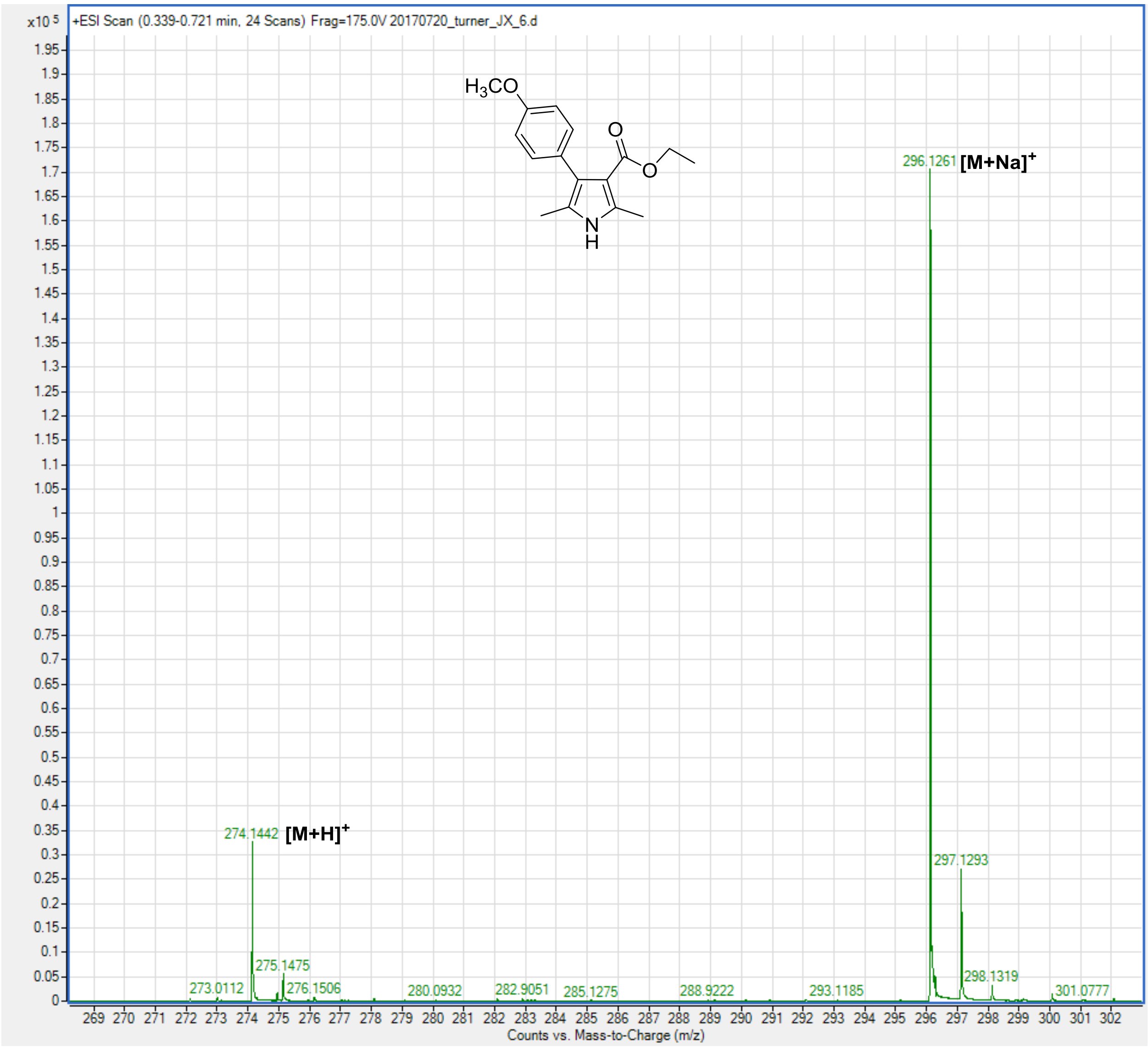


**Figure S27**: HRMS spectrum for Ethyl 2,5-dimethyl-4-(4'-methoxyphenyl)-1*H*-pyrrole-3-carboxylate (**11a**)

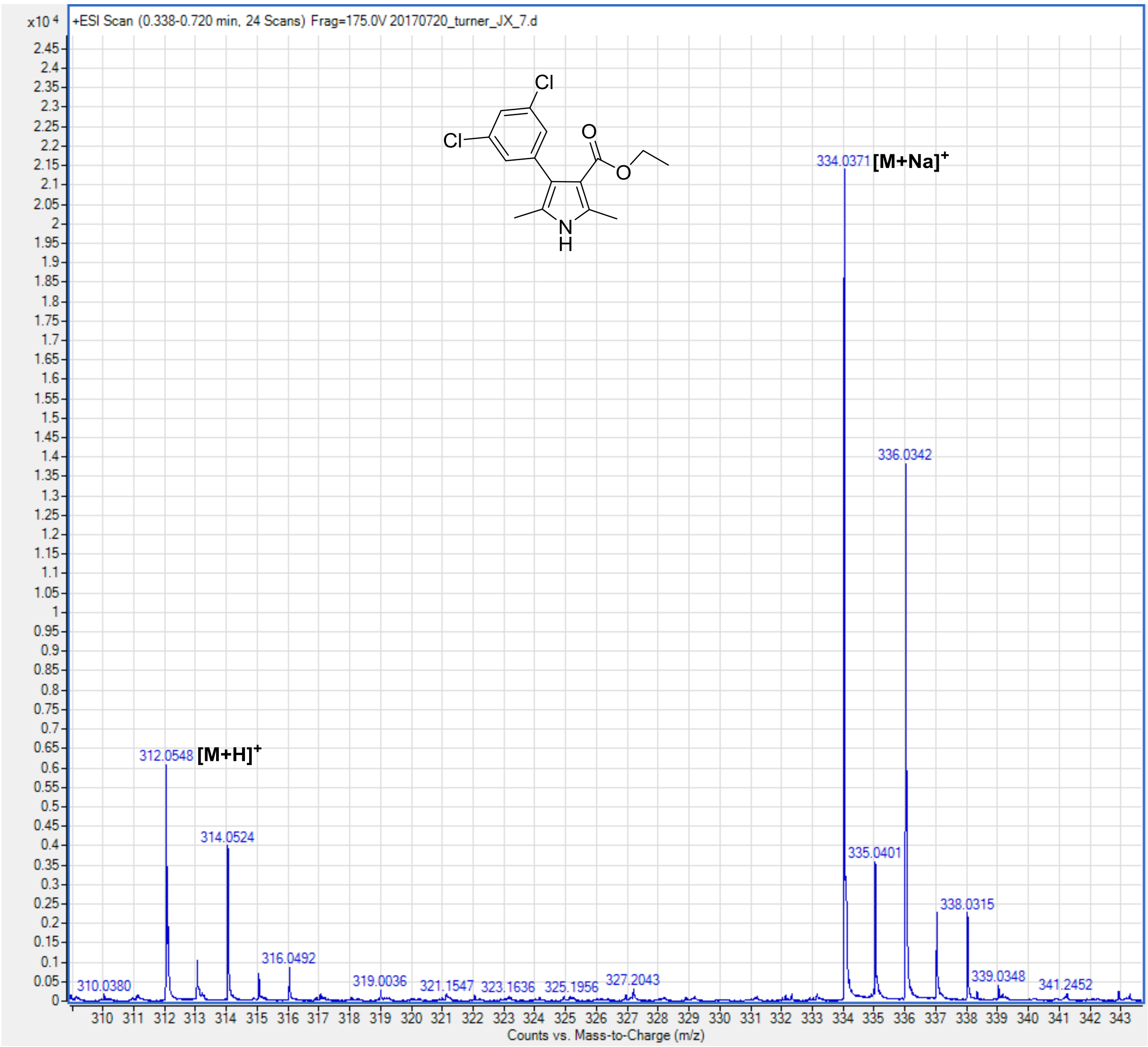


**Figure S28**: HRMS spectrum for Ethyl 2,5-dimethyl-4-(3',5'-dichlorophenyl)-1*H*-pyrrole-3-carboxylate (**12a**)

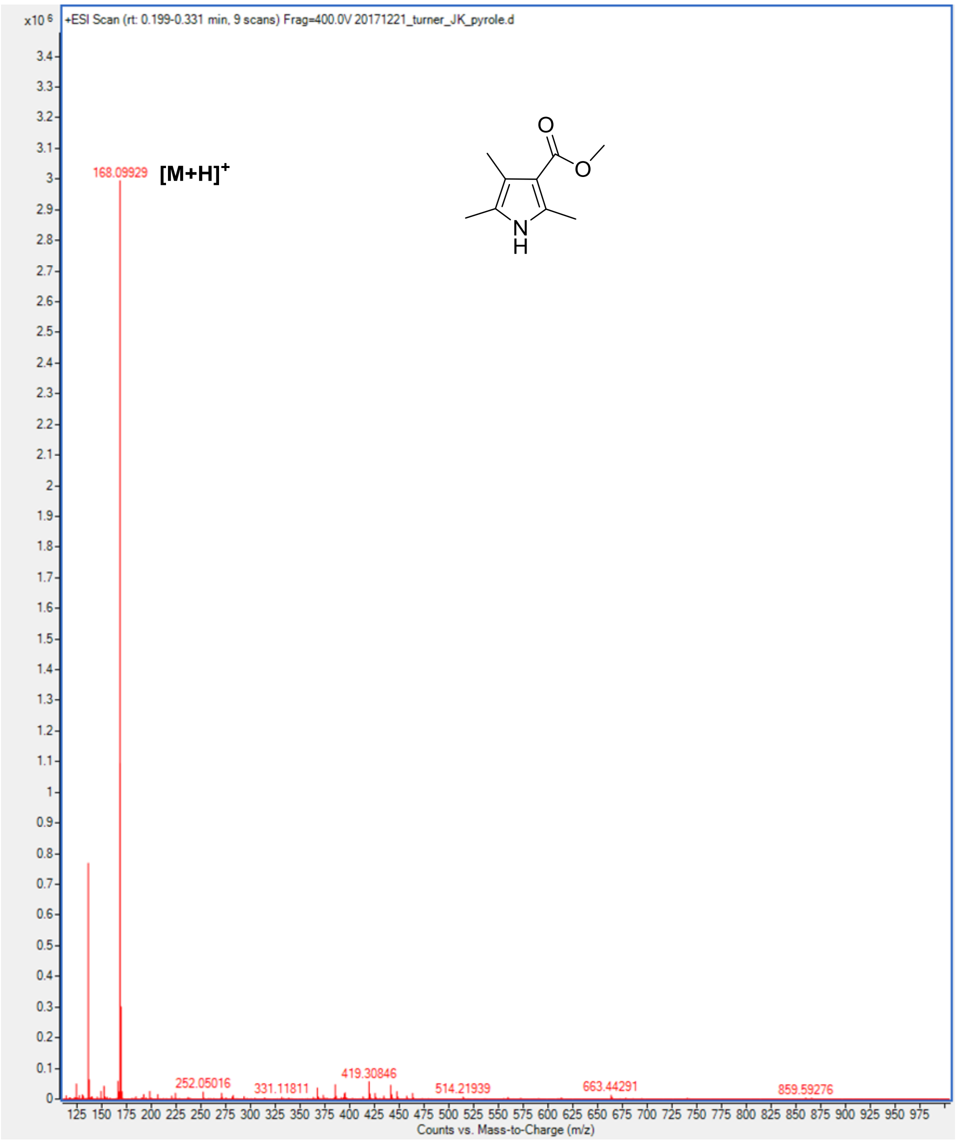


**Figure S29**: HRMS spectrum for Methyl 2,4,5-trimethyl-1*H*-pyrrole-3-carboxylate (**2b**)

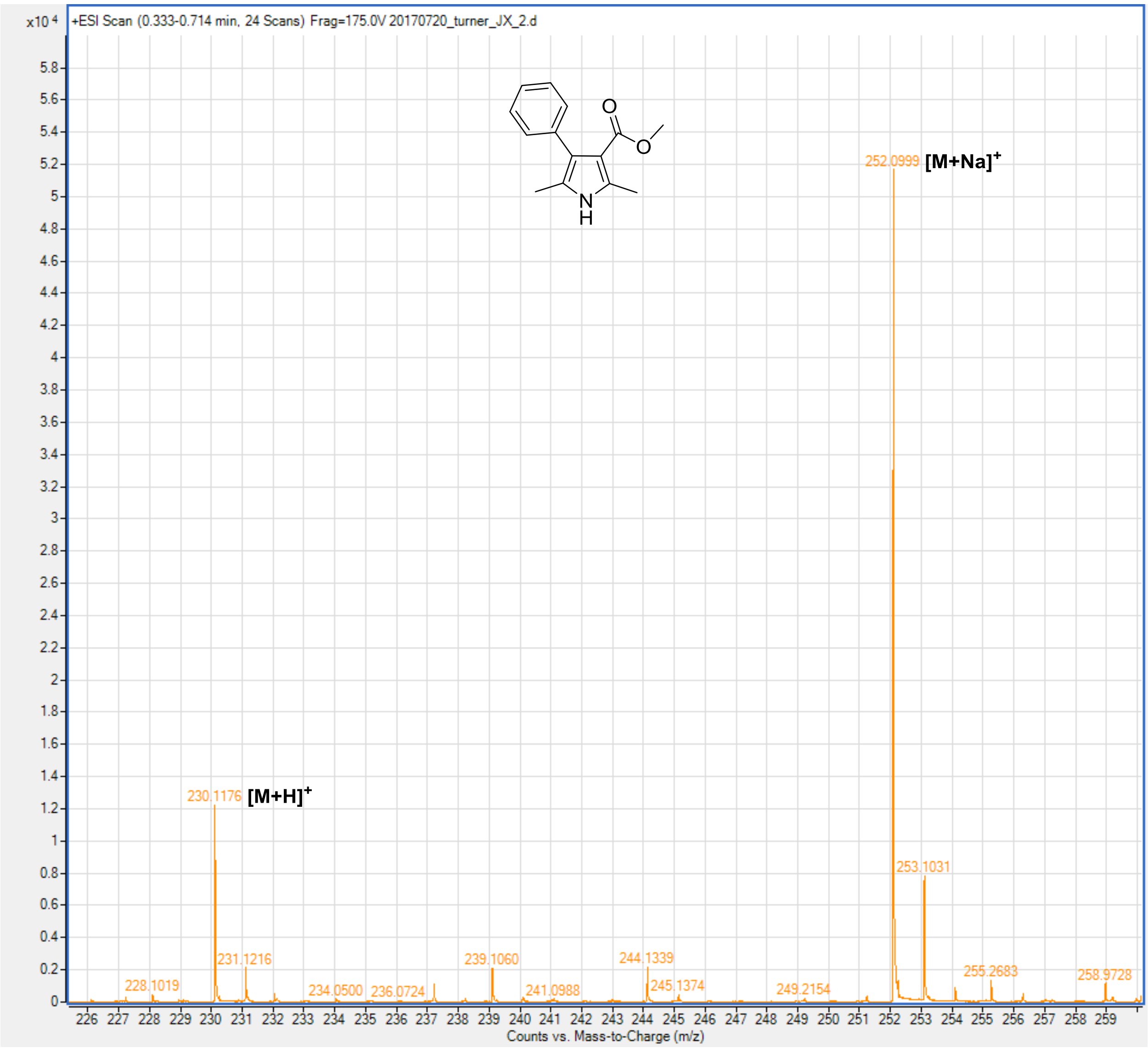


**Figure S30**: HRMS spectrum for Methyl 2,5-dimethyl-4-phenyl-1*H*-pyrrole-3-carboxylate (**7b**)
